# Fiber‐Format Flexible Linear Electrostatic Motors

**DOI:** 10.1002/adma.75156

**Published:** 2026-09-27

**Authors:** Sylvain Schaller, Herbert Shea

**Affiliations:** ^1^ Soft Transducers Laboratory Ecole Polytechnique Fédérale de Lausanne (EPFL) Neuchâtel Switzerland

**Keywords:** electrostatic actuator, linear motors, soft robotics

## Abstract

Linear motion is fundamental to robotics, yet existing solutions are either rigid electromagnetic motors or contractile artificial muscles with limited stroke and trade‐offs in force versus displacement. We report a sliding motor in a thin fiber format: a flexible, electrostatic linear actuator with a stroke limited only by fiber length. Comprising two coaxial fibers with helically wound electrodes, this “FiberMotor” generates bidirectional, fast, stepping motion with stroke‐independent force and inherent back‐drivability, a key feature for safe and transparent human–robot interaction. By eliminating permanent magnets and Joule heating, it offers a compact alternative to traditional motors. Modular bundling allows scaling to higher forces. Supported by an analytical model, this silent, lightweight technology can enable large‐scale integration into textiles, providing a promising building block toward future soft exosuits and wearable assistive devices.

## Graphical abstract

A flexible coaxial fibre‐format electrostatic motor enables backdrivable and reversible linear actuation with high force density and functionality under bending, offering a scalable actuation platform for soft and wearable robotics.

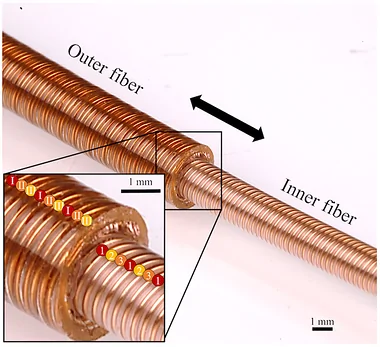

## 1 Introduction

Linear motion is foundational to robotic function, yet achieving it in wearable systems remains a significant challenge. Traditional linear actuation typically relies on rigid electromagnetic motors coupled with transmission mechanisms, which are often heavy, require permanent magnets, and consume power even when holding a static load. Backdrivability can be achieved by pairing low‐torque motors with low‐ratio planetary gearing, or by using much larger high‐torque direct‐drive motors.

Wearable robotics, such as soft exosuits [1, 2, 3, 4, 5, 6, 7, 8], implantable medical devices [3, 9], and wearable kinesthetic haptic interfaces [10, 11, 12, 13], require actuators that provide distributed force without restricting human motion. These actuators must be lightweight, bidirectional, functional when bent, and backdrivable for safe operation [14, 15]. Fiber‐format actuators are emerging as a promising solution, as their thin geometry allows them to be woven directly into flexible textiles and bundled into muscle‐like assemblies that maintain conformal contact with the human body.

In contrast to the continuous rotation of electromagnetic motors, most artificial muscles undergo structural deformation rather than relative displacement; that is, they elongate [16, 17], contract [18, 19, 20], twist [20, 21, 22, 23], or bend [24, 25], in response to external stimuli. This shape change limits the stroke and leads to a generated force that drops to zero at the maximum stroke. In addition, many of these compliant actuators require pre‑stretching or a pre‐load, which makes integration challenging, and provide only unidirectional motion unless used in an antagonistic configuration.

Electric‑field‑driven [26, 27] approaches to fiber‐format actuators include dielectric elastomer actuator (DEA) fibers [16, 28, 29]. While soft and stretchable, energy densities below 1 kJ/m^3^, actuation strains less than 10%, and force output is limited due to the low Young's modulus of the elastomer. Electrochemical yarn actuators [23] based on multi‑walled carbon nanotubes generate axial contraction with high specific power (61 W/kg at 5 V), but the strain is only ∼1%. Peano‐HASELs [27] offer appealing specific force and power, but have not been fabricated in fiber format.

Thermally actuated devices include liquid‑crystal‑elastomer fibers [18, 30, 31], which provide large strain (60%) with work densities exceeding 20 kJ/m^3^. Shape‑memory alloys [20, 32, 33, 34], shape‐memory polymer [35], and twisted‑and‑coiled polymer fibers [19] deliver large strokes and high specific energy (>2.6 kJ/kg), but their efficiency is low (∼1%). The cycling speed of thermal actuators is constrained by cooling times, making them far too slow for many wearable applications.

Fluidic fiber actuators [17, 22, 36, 37, 38], including hydraulically amplified filaments and McKibben actuators, achieve strains from 20% to 250% through pressure‑induced elongation or untwisting, but require pumps, valves, and high‑pressure sources that limit portability. A quantitative comparison of these actuator classes is provided in Table S1, summarizing motion type, stroke range, speed, and operating principles.

**TABLE 1:** FiberMotors pulling and holding forces for different dimensions. This table presents the measured pulling and holding forces, along with the unscaled values predicted by the 2D analytical model.

A	ID	A1	A1	B1	B2	C3
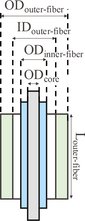	Measurement method	Clamped	Payload	Payload	Payload	Payload
	L_outer‐fiber_ (mm)	40	40	70	83	115
	D_copper wire_ (mm)	0.235	0.235	0.185	0.185	0.145
	OD_core_ (mm)	1.75	1.75	1.75	1.1	0.7
	OD_inner‐fiber_ (mm)	2.28	2.28	2.18	1.55	1.06
	ID_outer‐fiber_ (mm)	2.4	2.4	2.4	1.8	1.2
	OD_outer‐fiber_ (mm)	3.24	3.24	3.16	2.5	1.86
Measured forces	Pulling force (mN)	Min: 100 Max: 115	132	458	112	213
	Holding force (mN)	Min: 300 Max: 330	247	750	279	230
Measured forces per unit length	Pulling force/Length (mN/mm)	2.5	3.3	6.5	1.3	1.8
	Holding force/Length (mN/mm)	8.2	6.2	10.7	3.4	2
Analytical model	Model. pulling force (mN)	50	50	100	80	70
	Model. holding force (mN)	343	343	465	370	256

Planar electrostatic sliding motors [39, 40, 41, 42] provide bidirectional actuation and enable very long strokes, but exist in a flat ribbon rather than fiber format. These thin sliding motors use high‐density electrode arrays to generate tangential Maxwell stresses, enabling precise step‐by‐step translation between a stator and a slider. They allow bending only on 1 axis, a major limitation for wearables.

A proof of concept of a three‐phase electrostatic actuation using helically wound wire electrodes was demonstrated in 11 cm‐long rigid prototypes in the 1990s in Japan [43]. Here we reimagine this principle as a flexible coaxial FiberMotor (Figure 1) that operates when curved on two axes and can be fabricated in a continuous process at meter‐long scale, key elements for applications such as wearables.

**FIGURE 1:**
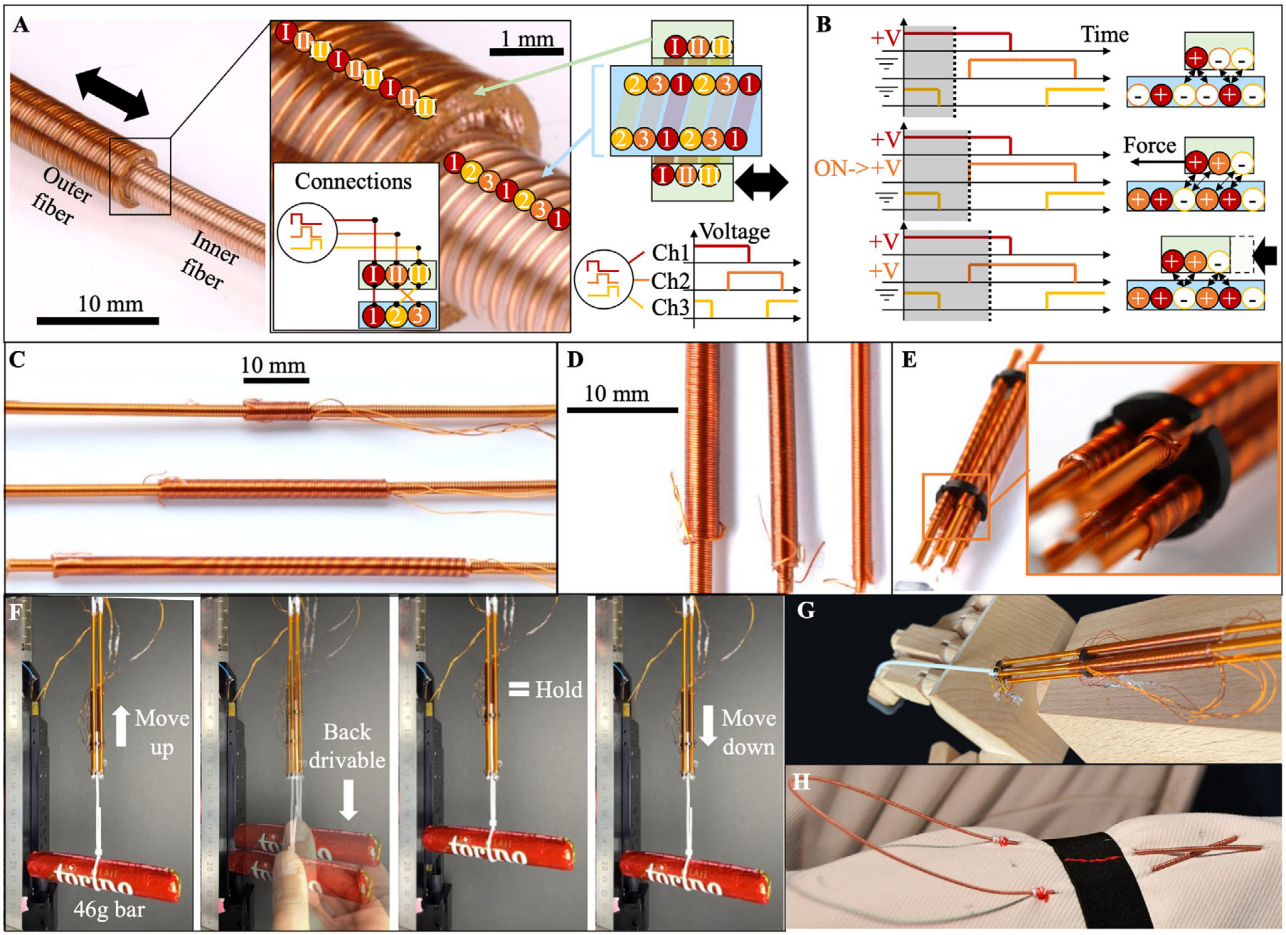
Fiber‐format linear electrostatic motor (FiberMotor). (A) The FiberMotor consists of two sliding concentric fibers, each consisting of a polymer shell encasing three helically wound insulated electrodes (here labeled I, II, and III for the outer fiber; 1, 2, and 3 for the inner fiber). The inner and outer electrodes are electrically connected to each other as schematically illustrated. The FiberMotor thus has only three wires extending from it, driven by a three‐channel unipolar power supply with a tunable phase offset. (B) The FiberMotor moves by one step when the voltages change. (C) Longer overlap between inner and outer fibers leads to higher force. (D) The winding fabrication process allows for different diameters. (E) Four FiberMotors are bundled in parallel to quadruple force output. (F) The motor is bidirectional, back‐drivable, and can hold a fixed position against a load, here raising a 46 g chocolate bar. (G) The motor is compliant, flexible, lightweight, and suitable for wearable applications (H) and for textile integration.

The FiberMotor actuator consists of two flexible coaxial fibers, each with three helically wound wire electrodes (Figure 1A). When driven by three‐phase square‐wave high‐voltage signals, electrostatic interactions between electrodes between inner and outer fibers produce an axial force generating relative motion between the two fibers (Figure 1B). By utilizing a sliding principle rather than material deformation, the FiberMotor maintains a constant force profile throughout its stroke and operates without the need for permanent magnets or the heat generation associated with Joule heating. The cylindrical design ensures passive coaxial self‐alignment and allows continuous, bidirectional, and back‐drivable motion (Figure 1F). Stroke is limited only by the fiber length. The actuator's fiber geometry, with outer diameters ranging from 1 to 3 mm, allows safe bending to mm radii of curvature and operating when bent to radii of curvature of a few centimeters.

The FiberMotors are characterized in detail for force, speed, and stroke as a function of drive conditions and geometry. An analytical model is reported that allows predicting performance as a function of material properties and geometry. Our devices have forward and backward no‐load speeds of over 85 mm·s^−1^, axial holding forces exceeding 750 mN per fiber, specific force over 450 N/kg, force density over 750 kN/m^3^, energy density over 5 kJ/m^3^ (active‐region mass/volume). For completeness, the corresponding work density computed using the total actuator volume is also reported in the Supporting Information. We use the active‑region volume because it contains all components that generate force and work (outer fiber and associated length of inner fiber). The inner fiber can be made longer or shorter without affecting speed or force. Stroke is limited only by the fiber length. The mass of the inner or outer fiber is 110 mg per cm.

The generated force is proportional to the overlap length between inner and outer fiber (which we call the “active region”). The fiber format facilitates integration into wearable systems and soft robotics, where thin, silent, and efficient actuation is critical. Although the devices we report here were tethered to a mains‐powered high‐voltage supply, the actuator's low current draw and minimal thermal footprint make it compatible with future embedded electronic systems. An overview of the FiberMotor and its operating principle is illustrated in Video S1.

During operation, a thin film of silicone oil between the inner and outer fibers prevents dielectric breakdown and reduces interfacial friction (Video S2). FiberMotor stroke, force, and speed can be tuned by adjusting dimensions (e.g., electrode pitch, fiber diameter; Figure 1C,D) and electrical drive conditions (e.g., voltage, frequency, and phase). The actuator operates when bent to a small radius of curvature, and the inner fiber can bend even more tightly than the outer. The number of wires and phases can be made larger than three to obtain smoother force curves. Due to its coaxial geometry, the achievable stroke is limited only by the total fiber length (Figure 1G). Multiple actuators can be assembled in parallel bundles to increase total output (Figure 1E). FiberMotors are a scalable, textile‐compatible platform for artificial muscles in wearable robotic systems (Figure 1H).

## 2 Results

### 2.1 FiberMotor Operating Principle and Fabrication by Winding

The electrostatic operating principle of the FiberMotor is conceptually similar to that used in planar‐shaped linear electrostatic motors [40, 41, 42], in MEMS‐based electrostatic steppers [44], and in three‐phase wire‐wound electrostatic actuators reported in the 1990s [43]. Prior analyses of different drive signals for planar structures exist, and the present analysis extends them to the co‐axial fiber geometry. Electrostatic forces pull offset parallel plates into alignment; periodically switching the voltages to the electrodes generates stepwise motion. Compared to planar devices, the flexible fiber factor allows for higher performance, longer stroke, easier electrical connections, and continuous fabrication.

As shown in Figure 1A, the FiberMotor consists of two concentric sliding fibers. The outer fiber has three helically wound wires (labeled electrodes I, II, and III) within an elastomer shell. The inner fiber consists of three helical wires (electrodes 1, 2, and 3) wrapped around an elastomer core. Electrical insulation between wires is primarily provided by the enamel coating on the wires. The inner electrodes are additionally coated with a high‐permittivity dielectric. Most of our experimental results were taken on 3‐phase motors; using more wires (e.g., 5) smooths the force‐versus‐position envelope, thus making the pulling force nearly equal to the holding force, at the expense of needing more drive phases.

Only the wires of either the inner or the outer fiber are connected to the power supply, because the electrodes of the inner and outer fibers are connected to each other as follows: I with 1, II with 3, and III with 2 (Figure 1A and Figure S1). The FiberMotor is driven by a three‐channel unipolar power supply that outputs square waves with a tunable phase offset and frequency, controlling speed and direction.

The step‐by‐step electrostatic sliding is schematically illustrated in Figure 1B for a simplified 2D cross‐section. Initially, channel 1 is at voltage V and channels 2 and 3 are grounded; all electrostatic forces cancel out, and there is no propulsive force. When channel 2 switches from ground to V, the new charge distribution generates electrostatic forces that lead to a net axial force. The fibers move by one step relative to each other, and the system then reaches a new stable equilibrium. Sequentially changing the voltages creates a stepwise motion along the fiber axis. The direction of motion is set by the voltage sequence. Speed is set by the switching frequency. The minimum number of wires that allows for bidirectional actuation is three; most of the FiberMotors we present are a three‐wire design. We also report four‐electrode designs. Using more electrodes can improve performance by providing a more continuous force profile.

The electrostatic attraction between the inner and outer helical electrodes occurs along the fiber overlap length. Each helical segment can be approximated as a curved parallel‐plate capacitor. To first order, the force scales with the electrode area, the permittivity of the dielectric, and the square of the applied electric field. Increasing the overlap between inner and outer fiber linearly increases the axial force (Figure 1C). The overlap region is the length of the shorter of the two fibers. Reducing the electrode diameter decreases the surface area per turn but increases the helix density, thereby increasing the capacitance per unit length (Figure 1D). Multiple fibers can be assembled in parallel bundles, enabling straightforward scaling of the force (Figure 1E).

Our experimental results demonstrate the design's versatility across stroke, scale, and force generation. Long‐stroke actuation exceeding 100 mm was achieved by combining 10 mm‐long outer fibers with 120 mm‐long inner fibers. Fabrication scalability was confirmed through miniaturization, with outer diameters reduced from 3.2 to 1.8 mm. Force scaling was validated through parallel assembly: a single 40 mm‐long, 3.2 mm‐diameter motor produced over 120 mN of pulling force, while a four‐motor bundle generated over 500 mN of pulling force. With work densities of 5.1 kJ m^−3^ (for the active‐region volume), these fiber‐format electrostatic motors approach the performance of human skeletal muscle (on the order of 10 kJ m^−^^3^), establishing a scalable architecture for high‐efficiency artificial muscles.

Figure 1F shows the FiberMotor raising and lowering a 46 g chocolate bar, holding a fixed position, and sliding when a person pulls hard enough against the drive direction. When the outer fiber is the mobile element, the maximum stroke is given by the length of the inner fiber. Figure 1G illustrates an example of motors used for future on‐body applications such as haptic feedback, assistive wearables, or rehabilitation. Figure 1H shows an example of motors integrated inside the textile of a pair of pants, illustrating that the motor can conform to curved geometries such as the knee or other parts of the human body and can be easily embedded into fabrics.

The fibers are fabricated using a filament winding process, inspired by one used for making EHD FiberPumps in our research group [45, 46]. As illustrated in Figure 2A, fabrication begins by winding enameled copper wires (140–230 µm diameter) around a central mandrel (Figure 2B and Video S9). For the outer fiber, the wires are wound around a steel rod, which is later removed, whereas for the inner fiber, the wires are wound on a thermoplastic polyurethane (TPU) filament core, which is part of the inner fiber. After winding, a dielectric layer is applied: the outer fiber is insulated by twisting TPU filaments around it and thermally fusing them. The inner fiber is dip‐coated with a 30 µm layer of poly(vinylidene fluoride‐co‐hexafluoropropylene) (PVDF‐HFP), a high‐permittivity dielectric. The steel mandrel is removed from the outer fiber; the inner fiber is then slid into the outer one, with an average inter‐fiber gap ranging from 60 to 120 µm. The inter‐fiber space is filled with a dielectric oil (Silicone oil 378348, Sigma–Aldrich, 20 cSt). The oil is held in place primarily by surface tension within the 60 to 120 µm inter‐fiber gap. Some leakage occurs initially after oil is applied using a pipette; then the motor operates for several hours before more oil is needed. The two fibers are finally electrically connected to each other, and either the inner or outer fiber is attached to the high‐voltage 3‐channel power supply. A cross‐sectional view of the assembled fiber motor, showing the materials, dimensions, and inter‐fiber spacing, is presented in Figure 2C.

**FIGURE 2:**
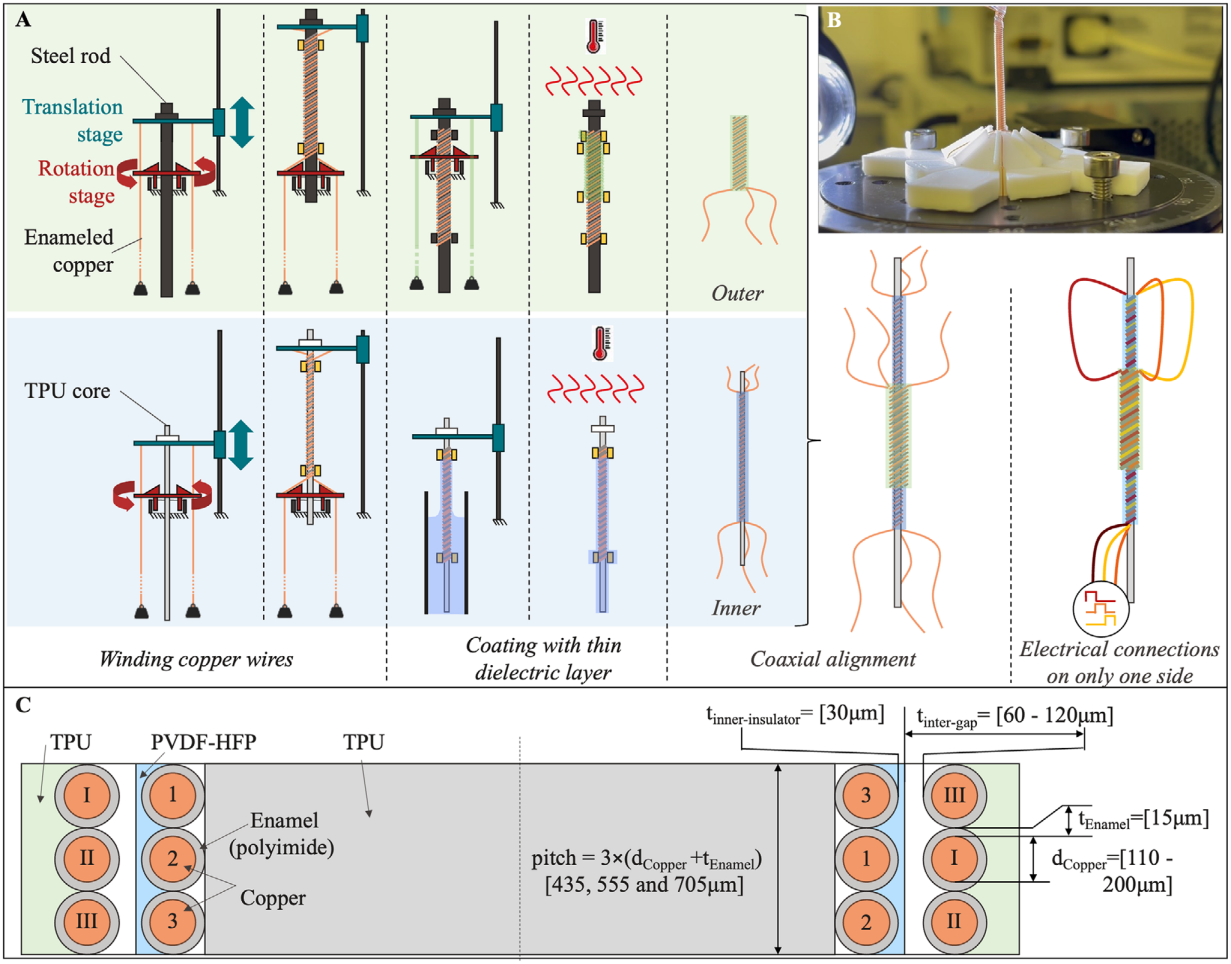
FiberMotor Fabrication process. (A) The fabrication process begins by winding enameled copper wires around a steel rod for the outer fiber and around a TPU core for the inner fiber. A thin dielectric layer is added, consisting of wound TPU for the outer fiber and dip‐coated PVDF‐HFP for the inner fiber. The TPU is fused at 180°C, and the PVDF‐HFP is cured at 105°C. The steel rod is removed from the outer fiber, and the inner fiber is inserted into the outer fiber. The electrodes of the inner and outer fibers are connected to each other at one end, while the electrodes at the other end of the inner fiber are connected to a 3‐channel power supply. (B) Photo of the rotation stage during the fabrication of the inner fiber. (C) Cross‐sectional view of the motor with materials and dimensions.

For the FiberMotors we report here, the winding length was limited by the length of the linear motor in the winding tool. Using 235 µm diameter copper wires and a 1.75 mm TPU core, the maximum winding lengths are 150 mm for the inner fiber and 90 mm for the outer fiber. Winding processes are suited to producing much longer fibers. We have developed a winding tool for EHD fiberpumps capable of making 9 m long fibers; this tool could readily be adapted to FiberMotors. By using different core diameters and wire diameters, we fabricated FiberMotors with different sizes, with pitches from 435 to 705 µm (see the Methods section). Typical masses for the inner or external fiber are 1.1 g per 10 cm segment.

### 2.2 Analytical Model of Force Versus Relative Position

We developed an analytical model that computes the static axial and radial forces as a function of relative fiber displacement. The motion of the fiber, pulling force, and blocking force can then be determined by overlaying the plots of force versus relative displacement for different drive conditions. To allow for a rapid exploration of the design parameter space and drive conditions without the computational overhead of finite element methods (FEM), we employed a two‐dimensional approximation of the helical structure, as shown in Figure 3A. Forces reaching beyond two electrode pitches, where one pitch corresponds to the spatial period of three electrodes along the fiber, are neglected. Charge injection phenomena and dielectric polarization effects are not included. Studies on the electrostatic forces in three‐phase sliding electrostatic structures have been reported in the 1990s literature [39, 40, 41, 42]; the present analysis extends this to the flexible co‐axial fiber geometry.

**FIGURE 3:**
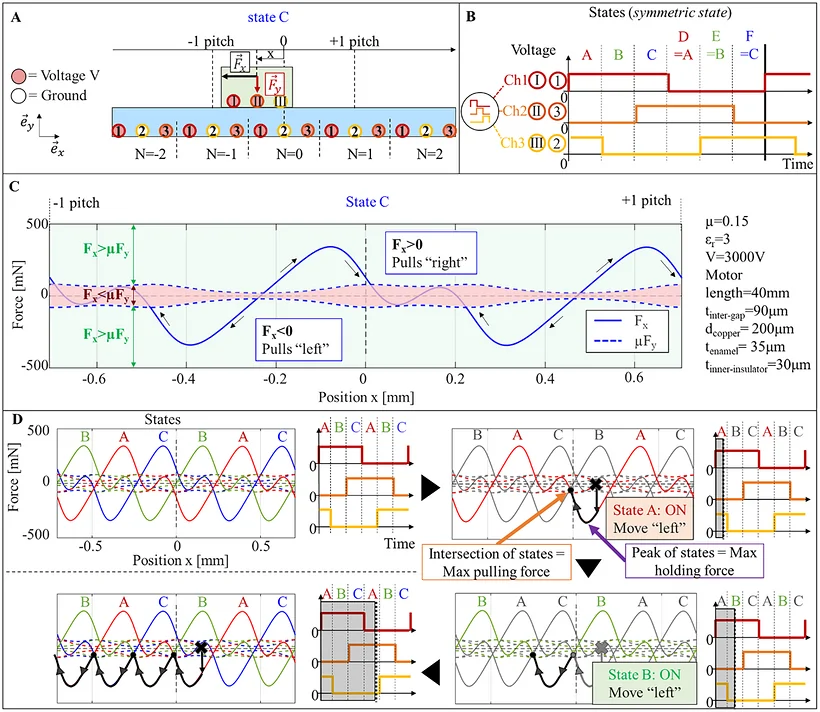
2D Analytical model to compute static propulsive and frictional forces. (A) 2D configuration used in the analytical model, consisting of a slider with three electrodes and a fixed base with 5 sets of three electrodes. (B) Square unipolar drive signal for each pair of electrodes: Channel 1–I/1, Channel 2–II/3, and Channel 3–III/2. (C) For one drive state (here we show state “C”), plot of propulsive and frictional forces *F*_x_ and µ*F*_y_ versus slider position, from –1 pitch to +1 pitch. When *F_x_* > µ*F_y_* > 0, the slider moves to the next stable position for that drive state. (D) Top left: Overlaid forces for drive states A, B, and C. Top right and bottom row: step‐by‐step actuation sequence, showing slider motion as one cycles through the drive states.

The actuation signals are square unipolar waveforms, matching what we use in the experiments. The electrostatic actuation of the FiberMotor is thus governed by a set of discrete voltage configurations, which we refer to as drive states. Each state defines a unique combination of high‐voltage and grounded electrodes, which in turn determines the direction and magnitude of the resulting forces. Six distinct drive states (A–F) are possible, based on permutations of voltage applied to the three channels.

For clarity, Figure 3A illustrates the geometry under drive state C, which serves as a representative example to discuss the force model. In this configuration, electrode pairs I/1 and II/3 are at high voltage (shown in red), while III/2 is grounded (in white). The inner fiber (blue) remains fixed, and the outer fiber (green) is mobile. The outer fiber comprises one spatial pitch of three electrodes (I, II, and III), while the inner fiber extends over five pitches: one aligned with the outer fiber (*N* = 0), two preceding (*N* = −1, −2), and two following (*N* = 1, 2). Contributions beyond *N* > ±2 are neglected. The resulting forces are the axial component *F*_x_ and the radial component *F*_y_ acting on the outer fiber on the *e*_x_ and *e*_y_ axes.

The applied electric field generates an attractive electrostatic pressure between the electrodes, known as the Maxwell stress σ:

$$\sigma =\frac{1}{2}\varepsilon_{0}\varepsilon_{r}E^{2}=\frac{1}{2}\varepsilon_{0}\varepsilon_{r}{\left(\frac{V}{d}\right)}^{2}$$

where *ε*_0_ is the vacuum permittivity, *ε*_r_ the relative permittivity of the dielectric between inner and outer electrodes, *V* the applied voltage, and *d* the normal distance between inner and outer electrodes.

This simple parallel‐plate expression is used here only to intuitively introduce the physical origin of electrostatic attraction. The full field distribution in helical geometry is more complex due to the geometry and to having several materials with different dielectric permittivities.

For three wires, there are six electrostatic drive states (A–F). Because the Maxwell pressure does not depend on the polarity of the voltage, there are only three unique drive states (see Figure S1). For example, configuration A (Channels 1 and 3 at high voltage, Channel 2 grounded) is equivalent to configuration D (Channels 1 and 3 grounded, Channel 2 at high voltage). For the rest of the analysis, we will use the following drive sequence A → B → C → A → B → C, which then repeats.

Figure 3B illustrates the three‐channel unipolar waveform applied to the electrodes. Outer electrodes I, II, and III are paired with inner electrodes 1, 2, and 3 as follows: I–1, II–3, and III–2. The three outer electrodes are driven by a unipolar power supply delivering square signals with tunable phase offsets. The duty cycle is defined here as the fraction of one period during which a given phase remains at non‐zero voltage. Phase II–3 is shifted relative to phase I–1 by a given phase, and phase III–2 is shifted relative to phase II–3 by the same phase.

The computed axial force *F_x_* versus position and the maximum frictional force µ*F_y_* versus position are plotted in Figure 3C for an imposed fixed displacement spanning –1 to +1 pitch. For the outer fiber to move, *F*_x_ must exceed the frictional force µ*F_y_*, where *µ* is the coefficient of friction between the fiber surfaces. Where the solid line (*F*_x_) lies within the dotted lines (± µ*F_y_*), friction dominates, and no motion will occur. When (*F*_x_) lies outside this band, the outer fiber can slide: a positive *F*_x_ drives motion in the positive axis direction, while a negative (*F*_x_) drives motion in the negative direction.

Drive states A and B are determined similarly to state C, representing lateral shifts of one‐third of a pitch (Figure 3D). With a 50% duty cycle and 120° phase shift, the sequence A → B → C → A → B → C generates a stepwise displacement of the outer fiber relative to the inner fiber. In Figure 3D (bottom left pane), starting from an arbitrary position, drive state A is applied. In this example, *F_x_* < µ*F_y_* < 0, so the outer fiber moves leftward until equilibrium is reached at the position where *F_x_* = µ*F_y_*. Drive state B is then activated, moving the fiber 1/3 of a pitch leftward. State C then generates the same motion, and the sequence repeats. No position sensing is required to set the appropriate voltage state, as long as the fiber can move by 1/3 of a pitch before the next change of voltage.

The intersections of the force versus position curves for different states define the pulling force *F_pull_*, which corresponds to the minimum force the fiber can exert on a load. The force developed by the fiber will vary as the fiber moves, and it will not drop below *F_pull_*. The peaks of the force versus position curves define the holding force *F_hold_*, which represents the maximum force before the fiber slips (i.e., the blocking force). A detailed representation of a simulated displacement cycle is shown in Figure S2.

The analytical model provides a predictive framework for evaluating the electrostatic forces that drive FiberMotor translation and determine the blocking force. The model shows how changing the order of the states enables bidirectional translation. These insights facilitate experimental interpretation and define critical performance limits, including pulling/holding capacity and step resolution.

### 2.3 Characterization of the FiberMotor

To validate the analytical model and characterize FiberMotor performance, we measured displacement speed and force generation across a range of drive conditions.

#### 2.3.1 Speed Characterization

The analytical model computes the electrostatic forces generated by each drive state, which determines whether the motor can overcome friction and initiate motion. Once this threshold is crossed, the motor advances by one step per cycle.

Setting aside inertial effects for now, the displacement speed is simply the product of the electrode pitch and the drive signal frequency *f*. A 120° phase shift generates forward motion, and a 240° phase shift generates backward motion (Figure S3). Each cycle of the drive signal produces six electrostatic drive states (A→B→C→A→B→C), corresponding to two helical pitches per signal period (Figure 4B). The theoretical velocity is therefore:

$$v_{theory}=2\times pitch\times f$$

**FIGURE 4:**
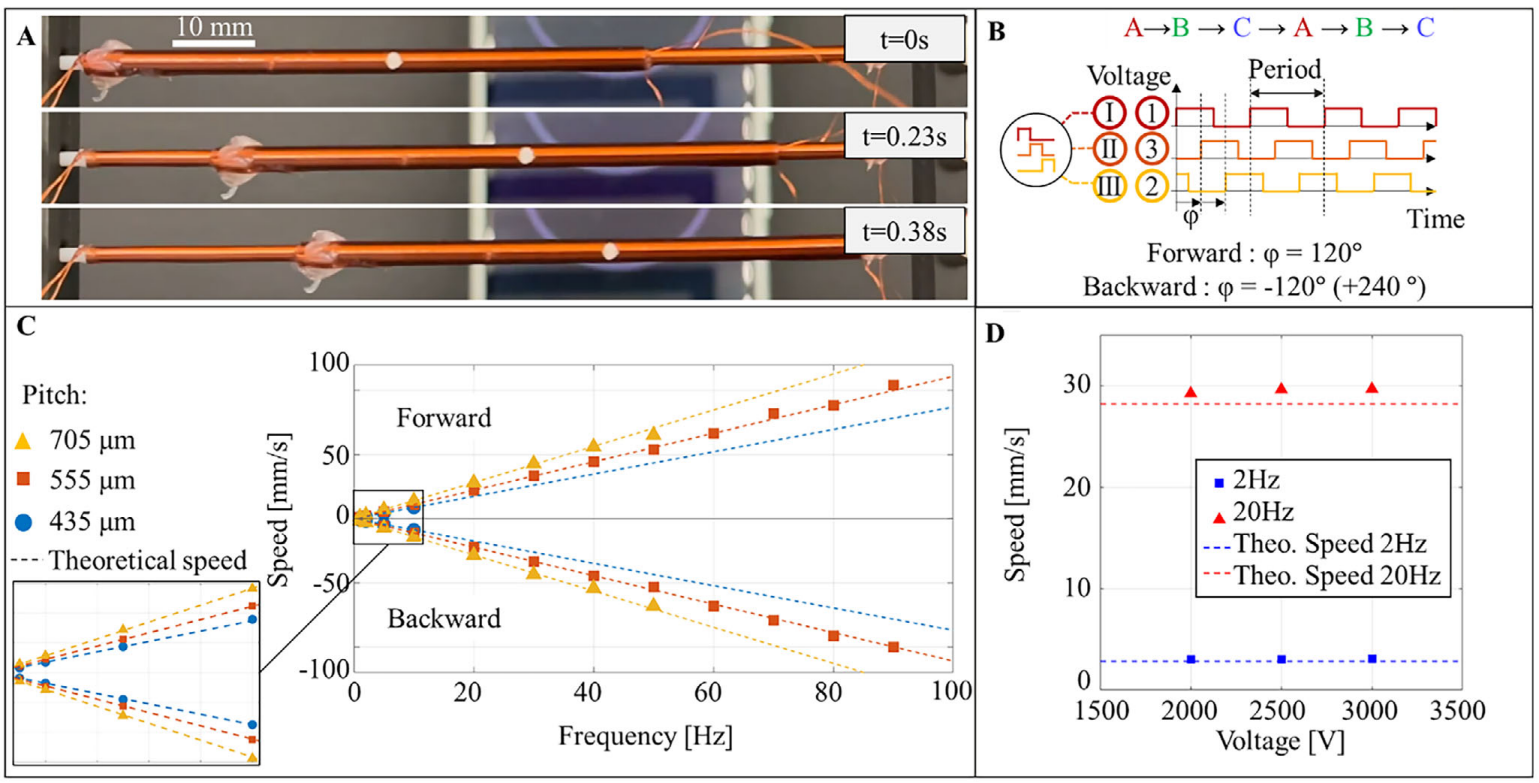
FiberMotor speed with no load. (A) The outer fiber moving relative to the fixed inner fiber at 3 kV at 80 Hz. (B) For *φ* = 120°, the outer fiber moves to the right; for *φ* = 240°, the outer fiber moves to the left. (C) Speed of the outer fiber as a function of drive frequency (markers) and predicted speed (dashed lines) for FiberMotors with pitches of 705, 555, and 435 µm. (D) Effect of drive voltage: above the voltage needed to overcome friction, speed is independent of drive voltage and depends only on drive frequency.

While the voltage influences the magnitude of electrostatic forces, it does not affect speed under no‐load conditions. When a load is applied (see payload characterization section), the motor may slow down or stall if the pulling force is insufficient to overcome friction and external forces.

The displacement speed was measured as a function of frequency *f* and applied voltage (Video S3 shows a FiberMotor driven at 80 Hz, with an average speed of 89 mm.s^−1^). Time‐stamped frames from the high‐speed recordings show the relative displacement of the outer fiber with respect to the fixed inner fiber (Figure 4A). The numbers we report are the average speed of the motor.

Measured speeds closely match the theoretical predictions across different pitch geometries (Figure 4C). In both forward and reverse actuation, the average displacement speed increases linearly with frequency. The motor with a 705 µm pitch reached a maximum speed of 65 mm s^−1^ at 50 Hz, the 555 µm pitch motor achieved 104 mm s^−1^ at 90 Hz, and the 435 µm pitch motor attained 9 mm s^−1^ at 10 Hz. For each device, the reported maximum speed corresponds to the highest frequency at which the motor produced continuous, uninterrupted motion; measurements showing discontinuities or missed steps were excluded.

To assess the influence of voltage, we tested the 705 µm pitch motor at 2.0, 2.5, and 3.0 kV, at both 2 and 20 Hz. The displacement speeds showed no dependence on voltage, confirming that, in the absence of load, speed is governed solely by frequency.

Above a certain frequency, the FiberMotor no longer moves smoothly, as shown in Figure S4. This limit comes about from dynamics, namely when there is insufficient time for the fibers to move a full step before the drive state changes. Inertial effects and friction both play a role. The power supply was not a limiting factor. Future work will explore design strategies to reduce friction and improve higher‐frequency performance.

#### 2.3.2 Force Characterization

We first performed static force measurements where fibers were held at a series of fixed positions while a single drive state (A, B, or C) was applied. This approach enables direct comparison with the analytical model (e.g., Figure 3C,D), which predicts force profiles based on electrode geometry and voltage configuration.

The inner fiber was attached to a force sensor while the outer fiber was mounted on a precision positioning system (Figure 5A). For each position, one drive state (A, B, or C) was applied, and the resulting force was recorded. The outer fiber was shifted in 5 µm steps until a complete pitch of 705 µm was covered (Figure 5B). Experimental data (circles) and the output of the analytical model (lines) are plotted for each drive state versus position in Figure 5D. The measured forces under static conditions with imposed relative position are approximately three times lower than predicted by the 2D model. To ease visual comparison, the model output was scaled down by a factor of 3.3. The shape of the measured static force profiles closely matches the shape of the analytical model. An unscaled version for a different FiberMotor is shown in Figure S5.

**FIGURE 5:**
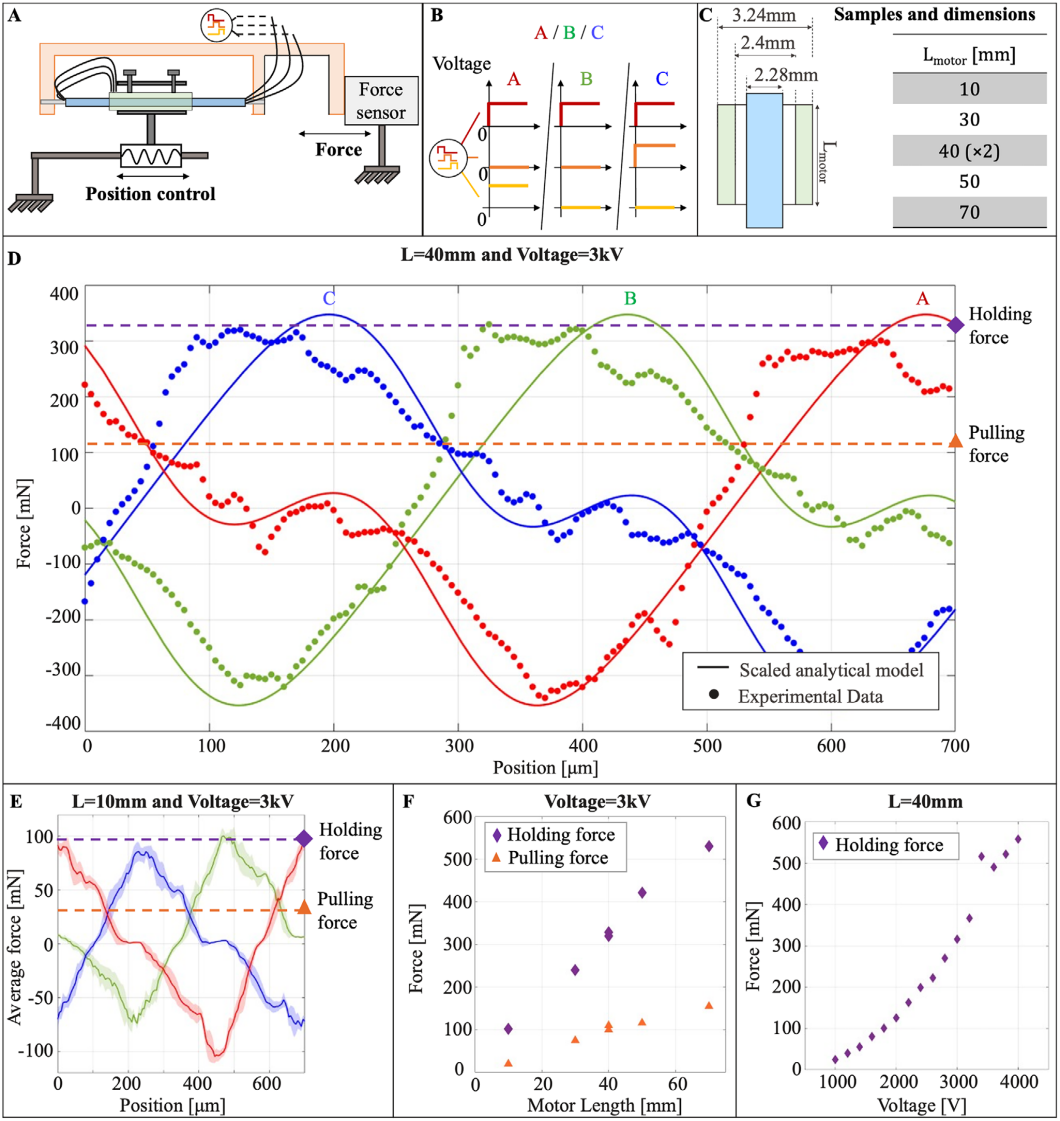
Measured force versus imposed position for different drive states and FiberMotor dimensions. (A) Schematic diagram of the test bench used for force characterization at fixed positions. The outer fiber is clamped to a motorized linear stage, while the inner fiber is clamped to a frame connected to a force sensor. (B) The three drive states (A, B, and C) are generated in sequence. (C) Six fiber motors were characterized, with outer fiber lengths varying from 10 to 70 mm. (D) Measured force versus position (data points) and scaled analytical model (solid lines) for the *L* = 40 mm motor at 3 kV, for drive states A (red), B (green), and C (blue). The analytical model was scaled down by 3.3 × to visually match the experimental data. (E) Force versus position for the L = 10 mm motor at 3 kV, averaged over moving by seven pitches. (F) Pulling and holding forces as a function of motor length at 3 kV. (G) Holding force as a function of voltage for a fixed drive state for the *L* = 40 mm motor.

From the force‐position curves, we can extract (i) the holding forces *F_hold_*, the periodic force maxima (here slightly more than 300 mN), and (ii) the pulling forces *F_pull_*, at the intersections between drive states (100 mN). The holding force is the maximum load the motor can hold at a fixed position, while the pulling force is the lowest propulsive force.

To assess repeatability along a fiber, the force–position relationship was characterized for a 10 mm motor over seven consecutive electrode pitches (i.e., for a 5 mm translation). The recorded data for each pitch were averaged; see Figure 5E. The shaded region represents the standard error. Figure 5E shows repeatable and periodic force generation as the fibers slide with respect to each other.

The effect of motor length on force output was evaluated by measuring holding and pulling forces for outer fibers of length 10, 30, 40 (two samples), 50, and 70 mm, all at a drive voltage of 3 kV; see Figure 5F. The inner fiber was made far longer than the outer fibers, so as not to be a factor in this experiment. The force output scales approximately linearly with the overlap length between the inner and outer fibers, from 102 mN for the 10 mm long outer fiber to 530 mN for the 70 mm long outer fiber. Similarly, the pulling force rose from 20 to 155 mN over the same range. The two 40 mm motors yielded consistent results, with holding forces of 320 and 329 mN and pulling forces of 100 and 110 mN.

In Figure 5G, we plot the holding force versus drive voltage of a 40 mm outer fiber actuated under drive state A at voltages from 1.0 to 4.0 kV in increments of 0.2 kV. The measured holding force increases from 25 mN at 1 kV to 558 mN at 4 kV. The 3 kV data (315 mN) was consistent with the holding force determined in the motor length study. The basic equation for Maxwell pressure predicts a quadratic increase in electrostatic force with voltage. Our measured force (Figure 5G) increases quadratically with *V* at lower voltages but shows a linear dependence above 2.4 kV. Several authors report non‐quadratic scaling of electrostatic force with voltage. For instance, Gravert et al. [47] observed that the electrostatic force in HASEL actuators also did not follow a simple quadratic dependence on voltage due to discharge hysteresis and nonlinear dielectric behavior. They introduced an apparent permittivity, a voltage‐dependent fitting parameter, that captures the effective dielectric response of the system under varying field strengths.

#### 2.3.3 Raising and Holding a Payload

We evaluate the performance of the FiberMotor under load, characterizing both the payload that can be lifted (“pulling force”) and the payload that can be held in a static position (“holding force”). In the lifting experiment (Figure 6A), the FiberMotor was mounted vertically and used to raise a load against gravity. The FiberMotor was activated in the forward direction for 20 s, then in the reverse actuation for 20 s (i.e., switching states A→B→C at 1 or 2 Hz). The displacement of the outer fiber was recorded using Kinovea motion tracking software; see Video S4.

**FIGURE 6:**
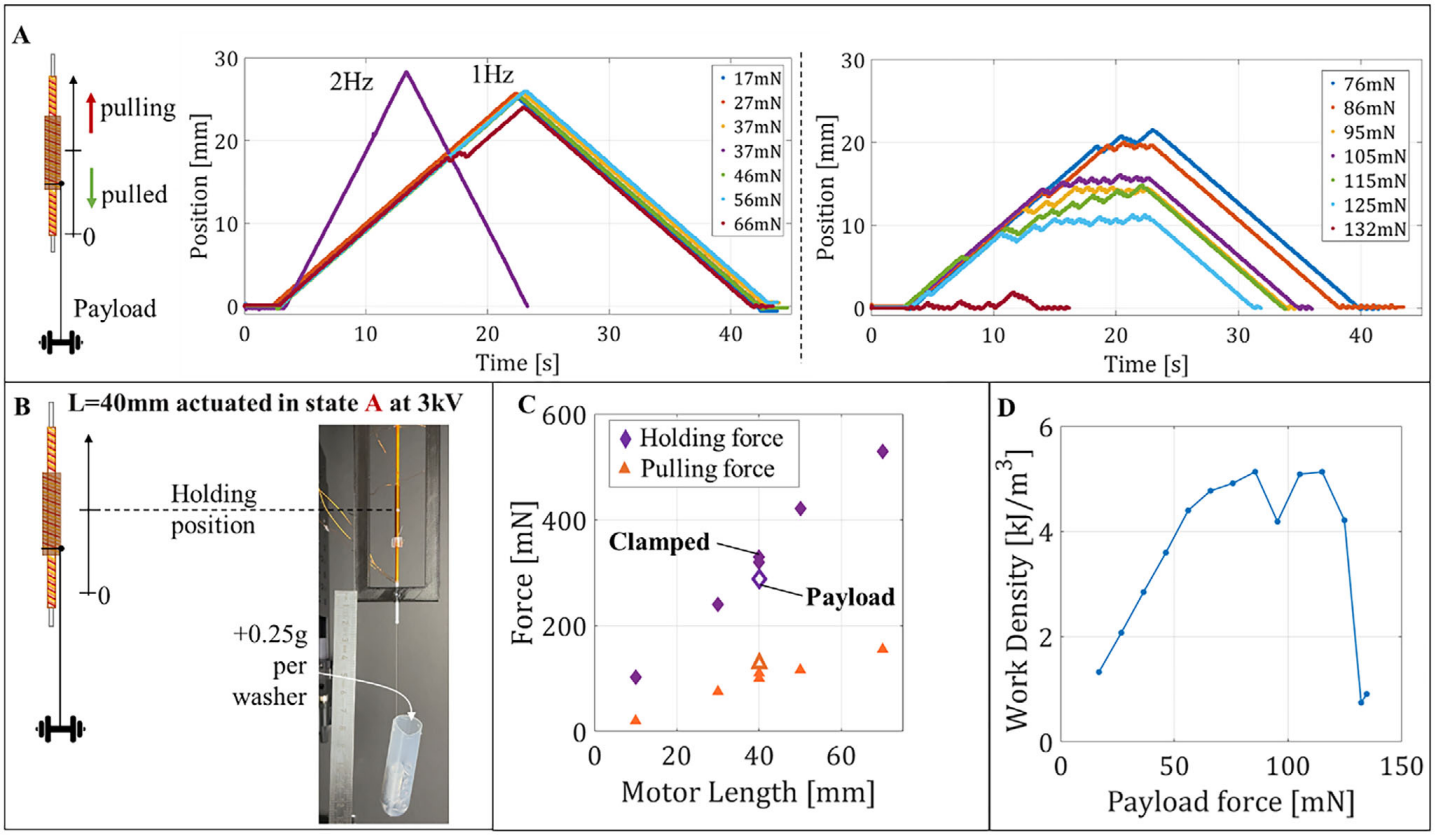
Raising and holding a Payload. (A) Vertical position versus time for a 40 mm‐long FiberMotor actuated at 1 Hz at 3 kV while raising and then lowering a payload. The 0.6 g mass of the external fiber is not included in the force and work calculations. The graph on the left is for payload masses from 1.7 to 6.7 g, for which the motor moves smoothly up and down; the graph on the right shows data for loads from 7.7 to 13.5 g, for which the motor is not always able to step reliably upwards, but where the downward speed is well controlled. To illustrate the effect of frequency on displacement, an additional measurement was taken at 2 Hz with a 3.8 g payload. (B) Schematic diagram and photograph of the “holding force” test setup. The motor was driven in drive state A. The payload was increased in 0.25 g steps until the outer fiber started sliding. (C) Force versus motor length as presented in Figure 5F, now including both pulling and holding force measurements for comparison under payload loading conditions. (D) Work density as a function of payload force.

In the holding test (Figure 6B), the outer fiber was actuated at drive state A and thus remained static. The payload was increased in 0.25 g increments until sliding occurred, identifying the maximum static “holding force” (Video S5).

All payload experiments were performed at 3 kV with a 40 mm long outer motor mounted around a much longer inner fiber. A driving frequency of 1 Hz gave an average speed of 1.4 mm/s. The displacement versus time plots recorded under different loads are presented in Figure 6A, where the left panel shows payloads from 17 to 66 mN, and the right panel from 76 to 132 mN. The purple curve corresponds to the 2 Hz actuation condition at 37 mN and illustrates the effect of increased frequency on displacement (simply doubling the speed). Continuous smooth forward and backward motion was observed for payloads up to 66 mN, with no missed steps. At higher payloads (>66 mN), intermittent displacement was seen: the FiberMotor did not always move forward when the drive state changed. The 66 mN threshold corresponds to the specific tested configuration and should not be interpreted as a general limit. This behavior reflects the open‐loop nature of our control system: in the absence of feedback, the motor simply continues to cycle through drive states even when the outer fiber shifts out of optimal alignment with the inner electrodes.

The FiberMotor is easily and safely back‐drivable: with no gears, when forced to move in the opposite direction in which it is driven, it simply slides with no mechanical damage. Once the outer overload is removed, the outer fiber re‐engages electrostatically with the inner fiber and resumes motion.

The static holding force results are summarized in Figure 6B and are compared to the clamped‐force data shown in Figure 6C. The measured holding force under payload was slightly lower than in the clamped configuration, while the pulling force was higher. We attribute this small difference to variations in how the fibers were mechanically constrained and actuated during the two experimental configurations. In the static tests, the motor was held at fixed positions with no motion, allowing pure force measurement under ideal alignment. In contrast, the dynamic tests involved continuous motion, where factors such as friction, inertial effects, and slight misalignment during actuation could influence the measured force. These differences likely account for the observed difference.

Work density was computed as a function of payload force using the volume of the outer fiber; details on the calculation are given in Supporting Information (Figure S6). Work density depends on the load, reaching a peak of 5.1 kJ/m^3^ at 115 mN (Figure 6D). The work density decreased sharply as the payload exceeded 125 mN, because the FiberMotor can no longer effectively move the payload.

Table 1 reports the measured pulling and holding forces for four FiberMotors (labeled A1, B1, B2, and C3), along with the corresponding values predicted by the unscaled two‐dimensional analytical model. Motor A1 is the largest device and was used for both clamped and payload characterizations. It is made with 0.235 mm diameter copper wire on a 1.75 mm diameter outer core. Motor B1 has the same core diameter (1.75 mm) but uses a smaller wire diameter of 0.185 mm. This motor has a specific force of 458 N/kg and a force density of 775 kN/m^3^, calculated using the mass and volume of the external fiber (1 g and 5.8 × 10^−7^ m^3^). Motor B2 retains the 0.185 mm wire diameter but reduces the core diameter to 1.1 mm. Motor C3 uses a smaller wire diameter of 0.145 mm and a core diameter of 0.7 mm.

Table 1 also reports the pulling and holding forces normalized by the length of the outer fiber. The pulling force per unit length ranges from 1.0 to 6.5 mN·mm^−1^, while the holding force spans 2.0–10.7 mN·mm^−1^. In all cases, the holding force is between 1 and 3 times greater than the pulling force, a trend that agrees with the analytical model. The scaling by 0.3 × of the analytical results in Figure 5 was to visually show the good agreement of the overall shapes of the force versus position profiles for motor A1 under static clamped conditions. The comparisons in Table 1, with no scaling factor applied, correspond to loaded, unclamped measurements with a payload attached, which probe a different frictional regime. These normalized force values are particularly useful for design in space‐constrained applications. Given a required force, the values allow estimating the necessary motor length or determining how many motors must be placed in parallel to meet the targeted force. This provides a practical framework for scaling the actuator architecture to match specific load requirements.

The analytical model predicts holding forces reasonably consistent with experimental values, but the model underestimates the holding force by a factor of two, highlighting the limitations of the model. These discrepancies arise primarily from the neglect of dynamic effects and the use of simplified approximations for friction.

Using the analytical model, we can predict how FiberMotor performance scales with geometry. For instance, reducing all fiber dimensions by a factor of 10 would theoretically increase force density by 100 times, assuming constant voltage. However, maintaining the same voltage at smaller scales would result in electric fields that exceed the dielectric strength of the material. One must rather work at a constant electric field, reducing the voltage as dimensions are reduced. Under this constraint, the model predicts a force density increase of about 10 times for a 10× reduction in dimensions. These scaling estimates provide a starting point for designing smaller motors not only for improved performance, but also for better integrability, enabling lighter, more flexible, and space‐efficient actuator systems.

#### 2.3.4 Operating When Curved

The inner fiber can easily be wrapped around a finger. However, the FiberMotor cannot operate at such a small radius of curvature. As an initial feasibility demonstration, we characterized the performance of FiberMotors on curved surfaces in view of wearable and robotic applications. The test setup is shown in Figure 7A, where the curvature of the FiberMotor can be accurately controlled. The FiberMotor moves smoothly up and down over its full 20 mm stroke when bent to a radius of curvature of 60 mm (Video S8). At smaller radii (i.e., at higher curvature), the added friction becomes too large for the FiberMotor to overcome. The radius of curvature for normal operation could be further reduced by decreasing the copper wire diameter, replacing the TPU encapsulation with a softer dielectric material, or shortening the outer fiber to reduce bending strain. The flexibility of the inner fiber could be improved by using a softer core filament, thinner copper wires, or more compliant encapsulation. The existing FiberMotor size is already flexible enough for basic integration in textiles (Figure 7B).

**FIGURE 7:**
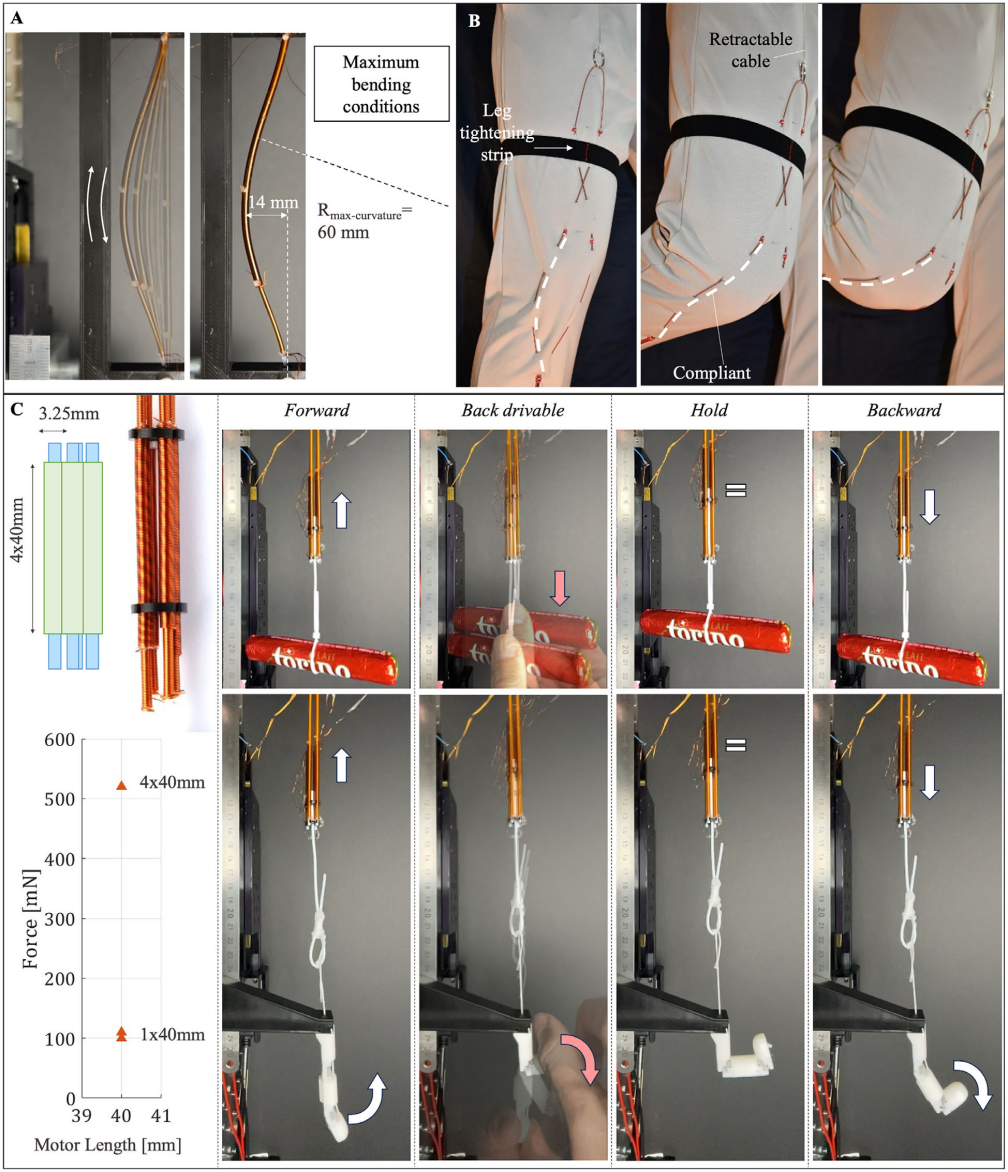
Operation of a FiberMotor when curved and of a bundle of FiberMotors. (A) The FiberMotor operates smoothly down to a radius of curvature of 60 mm. (B) FiberMotors integrated into textile, showing the actuator bending around a mannequin knee. (C) Parallel bundle of four FiberMotors. In one application (top row), the fiber bundle was used to raise a 46 g chocolate bar. In the second application (bottom row), the bundle actuated a tendon‐driven printed finger. In both cases, the motion was first forward‐driven, then manually pulled back to demonstrate back‐drivability, followed by a holding phase, and finally actuated in reverse to return to the original position.

Twisting, stretching, and other deformations were not characterized and may affect electrode alignment and electrostatic force. The current open sliding configuration can be influenced by external contact, and fully wearable integration will require an improved confinement solution.

#### 2.3.5 Bundling for Higher Force

To demonstrate modular scaling of force, we assembled a bundle of four FiberMotors, each with a 40 mm long outer fiber, mechanically linking them with 3D‐printed TPU connectors (Figure 7C). The four inner fibers were electrically connected in series, as were the four outer fibers (Figure S7). To complete the circuit, one inner fiber was electrically linked to one of the outer fibers, and another inner fiber was connected to the high‐voltage supply, enabling simultaneous activation of all four FiberMotors in the bundle.

We studied two application examples with the FiberMotor bundle. In a lifting scenario, the bundle lifted a 46 g payload (a chocolate bar, Video S6). In a robotic scenario, the bundle actuated a tendon‐driven printed robotic finger (Video S7). In both cases, the actuator displayed forward motion, backdrivability, static holding, and reverse motion.

The force output of the four‐motor bundle was compared against the single‐motor configuration. Based on previous measurements for two 40 mm motors (100 and 110 mN), the expected pulling force for a four‐motor configuration was 400–440 mN (Figure 7C). However, we measured a maximum pulling force of approximately 530 mN. This enhanced force may stem from the distributed nature of the load, though two‐ and three‐motor configurations were not tested.

## 3 Discussion

Robots rely on rotating sliding motors, almost always operating on electromagnetic principles. We present here a sliding motor in a flexible fiber format, combining the advantages of sliding systems (e.g., force that is independent of stroke) and of electrostatic drive (very low power consumption to hold a fixed position) with the fiber format of many artificial muscles. The FiberMotor achieves promising specific force while remaining silent, flexible, bidirectional, and back‐drivable. It bridges the gap between the high force of rigid motors and the mechanical compliance of fiber‐format actuators, without the stroke limitations or cooling requirements of traditional artificial muscles.

Compared to back‐drivable linear motors, the FiberMotor has high specific force, approximately 450 N/kg, and a force density of approximately 775 kN/m^3^. Given its flexible format, bending easily on 2 axes, FiberMotors are particularly well suited for textile integration. The coaxial geometry enables self‐alignment without rigid guiding components, allowing continuous translation and back‐drivability under curvature and load, key features for wearable systems. Stroke is only limited by the length of the longest fiber, enabling new architectures as strain is no longer a limiting factor, unlike the many types of fiber‐format artificial muscles. The FiberMotors we reported here, integrated in textiles, did not have an enclosure to prevent oil leakage. More robust integration in wearables will require validated oil confinement strategies or alternative dry operation approaches, as well as more extensive characterization under repeated bending. In addition, integrating high‑voltage actuators and their associated electronics on the body will impose constraints related to safety norms and regulatory requirements, which must be addressed in future system‑level designs.

We developed a static analytical model to guide the motor design. The model shows up to a factor of two over‑ and under‑prediction of absolute holding force across the four geometries in Table 1. Table 1 reports measurements with a payload attached. In Figure 5D, for a static clamped position, the model overestimates the force by 3×, but shows very good agreement in the shape of force versus position. The two test conditions lead to different effective frictional behavior, and hence the different agreement with the model. The friction coefficient *µ* was initially estimated from literature polymer properties and then empirically tuned to reflect the lubricating effect of silicone oil used during operation. The model assumes a simplified dielectric environment and does not account for properties of all materials. For instance, silicone oil alters the local electric field distribution and adds viscous damping, both of which influence static and dynamic response. Several effects central to electrostatic sliding actuation—friction, dielectric polarization, lubrication, gap‑dependent field distribution, long‐term charging effects, and dielectric breakdown phenomena—merit further investigation. FEM simulations could include the full 3D geometry, allowing for more accurate predictions by taking the full field distribution into account.

Performance was experimentally tuned by varying electrode pitch, drive voltage, and motor length, as shown in Figures 4, 5, 6, with the holding force of single fibers exceeding 500 mN and actuation speeds over 85 mm·s^−1^. Geometry and electrical parameters can be chosen to tailor actuator output across a range of configurations. Typical lifetimes were well beyond 1000 cycles, with some samples operating continuously for 24 h; the most common failure mode was dielectric breakdown.

Speed measurements were performed under no‑load conditions, and operation under load was characterized at low frequencies (1–2 Hz). A complete force‐speed‐power map under load, including actuator efficiency (input electrical power vs. output mechanical power), is identified as future work.

There remains substantial potential for higher specific force and power through the optimization of material properties, for example, employing liquid dielectrics with higher relative permittivity, thinner solid dielectric coatings, and using much smaller wire diameters to reduce weight increases both helix density and effective capacitance per unit length. The pulling force can be made nearly equal to the holding force by using more wires per fiber, because having more phases smooths out the envelope of the force versus position curves for multiple phases.

We operated the FiberMotor in open‐loop. While this configuration validates the motor's fundamental functionality, the lack of real‐time positional data prevents the system from correcting for missed steps when the actuator is subjected to payloads exceeding its pulling threshold. Applications requiring accurate sub‐mm positioning or autonomous adaptation to dynamic mechanical environments will benefit from future integration of sensing and closed‐loop feedback mechanisms.

A scaling analysis suggests that reducing fiber dimensions by a factor of 10 could increase force density by the same factor. This points to the need for new fabrication processes, for instance, advanced multi‐material winding methods, to manufacture bundles of 100 µm‐diameter FiberMotors for high‐stroke, high‐power‐density compliant actuators.

Future work will focus on integrating active sensing and feedback control, using dielectric coatings with improved breakdown strength and tribological properties, and exploring long‐term cyclic durability under load. These advances will support the deployment of fiber electrostatic motors as artificial muscle modules in exosuits, robotic garments, and lightweight assistive systems.

## 4 Materials and Methods

### 4.1 Informed Consent

Informed consent was obtained from all participants.

All experiments were performed under standard laboratory conditions (room temperature, ambient humidity), using the measurement setups and instrumentation detailed below.

### 4.2 Sample Geometries

Three FiberMotor geometries were used for speed measurements:

– 705 µm pitch, 40 mm long outer fiber, 120 mm long inner fiber, and 3.24 mm outer diameter.
– 555 µm pitch, 70 mm long outer fiber, 120 mm long inner fiber, and 3.16 mm outer diameter.
– 435 µm pitch, 115 mm long outer fiber, 120 mm long inner fiber, and 1.86 mm outer diameter.

Six FiberMotor geometries were used for force measurements:

– 705 µm pitch, 10, 30, 40, 40, 50, and 70 mm long outer fiber; 120 mm long inner fiber; and 3.24 mm outer diameter.

For scaling analysis (Table 1), the motors were:

A1: 0.235 mm diameter copper wire, 1.75 mm diameter core.

B1: 0.185 mm diameter copper wire, 1.75 mm diameter core.

B2: 0.185 mm diameter copper wire, 1.1 mm diameter core.

C3: 0.145 mm diameter copper wire, 0.7 mm diameter core.

### 4.3 Electrical Drive and Signal Configuration

The FiberMotors were driven by an Octo‐Channel High Voltage Power Supply (https://gitlab.epfl.ch/schouten/OCHVPS/‐/tree/v1.0) providing up to 4 kV square wave signals per channel at up to 2.5 mA current, with adjustable duty cycle and phase shift per channel.

A representative voltage and current trace for one phase at 1 Hz is provided in Figure S8, illustrating the typical electrical waveform used in this study, the current and power draw (instantaneous and average), and device capacitance.

### 4.4 Speed Measurements

Translation speeds were measured for drive frequencies from 1 to 100 Hz and voltages from 2.0 to 3.0 kV. The outer fiber displacement was recorded using an iPhone 12 Pro camera at 240 fps. Motion tracking was performed with Kinovea software. Tests were done at no load. Each test captured the complete bidirectional displacement of the outer fiber sliding along the inner fiber under periodic drive signals.

Three motors with different electrode pitches and geometrical configurations were evaluated. The first motor had an electrode pitch of 705 µm, a 40 mm‐long outer fiber, and an outer diameter of 3.24 mm. The second motor had a 555 µm pitch, a 70 mm outer fiber length, and an outer diameter of 3.16 mm. The third motor had a 435 µm pitch, a 115 mm outer fiber length, and an outer diameter of 1.86 mm.

Speed measurements were performed at a constant drive voltage of 3 kV over a range of switching frequencies: 1, 2, 5, 10, 20, 30, 40, 50, 60, 70, 80, 90, and 100 Hz. Frequencies for which no data are reported correspond to conditions where the actuator either did not move or produced non‐continuous displacement.

### 4.5 Force Characterization

Force measurements were performed by clamping the inner fiber to a vertical FUTEK LRF400 load cell (1.1 N range), connected to a FUTEK IPM650 controller. The outer fiber was attached to a Standa linear stage (2.5 µm resolution), enabling incremental displacements of 5 µm across one electrode pitch. Measurements were performed for drive states A, B, and C at 3 kV. Motor length was varied using outer fibers of length 10, 30, 40, 50, and 70 mm on a 100 mm long inner fiber.

At each measurement step, the outer fiber was held fixed, a drive state (e.g., A) was applied, and the resulting force on the inner fiber was recorded. The procedure was repeated for drive states B and C before shifting the outer fiber by 5 µm (Figure 5B).

### 4.6 Payload Characterization

Payload tests (lifting and holding) were conducted using 40 mm long outer fibers. Weights were attached via a nylon cable glued to a silicone tube fixture partially wrapped around the fiber. Displacement was tracked with Kinovea software during forward and reverse actuation at 1–2 Hz. For holding tests, washers were added in 0.25 g increments until slip occurred.

### 4.7 Bending Tests

For bending operation, motors were mounted in a custom frame, clamped at the top, with the TPU inner fiber fixed to a linear stage. Compressive displacements were applied in 100 µm increments, inducing progressive buckling of the FiberMotor. Forward and reverse actuation was tested at each step, up to a maximum lateral deflection of 14 mm, corresponding to a bending radius of 60 mm.

### 4.8 Bundle Assembly

Bundles of four motors were assembled using 3D‐printed TPU connectors. The fibers were electrically connected in series, with one inner fiber and one outer fiber connected to the power supply. The bundle was tested for lifting a 46 g payload and for actuating a robotic finger.

## Author Contributions

S.S. and H.S. formulated the concept. H.S. provided oversight and acquired the financial support. S.S. designed and fabricated the samples, built and automated the test bench, and performed the measurements. S.S. and H.S. analyzed the data. S.S. prepared the figures. S.S. and H.S. wrote the manuscript.

## Conflicts of Interest

The authors declare no conflicts of interest.

## Supporting information




**Supporting File 1**: adma75156‐sup‐0001‐SuppMat.pdf.

**Supporting File 2**: adma75156‐sup‐0002‐SuppMat.zip.

## Data Availability

The data that support the findings of this study are available from the corresponding author upon reasonable request.
