# Supplementary material for: Fiber‐Format Flexible Linear Electrostatic Motors
Source: Adv Mater. 2026 Sep 27;38(63):e75156. doi: 10.1002/adma.75156 (PMC13755658; doi:10.1002/adma.75156)
Supplement: Supplementary file 1 [file ADMA-38-e75156-s002.pdf]

Supplementary information

Fiber-Format Flexible Linear Electrostatic Motors

Sylvain Schaller and Herbert Shea

Soft Transducers Laboratory

Ecole Polytechnique Fédérale de Lausanne (EPFL)

Rue de la Maladière 71b, CH-2000 Neuchâtel,

Switzerland

Contents:

- Captions of SI videos S1 to S9
- SI Table 1
- SI Figures S1 to S8

SI-Videos

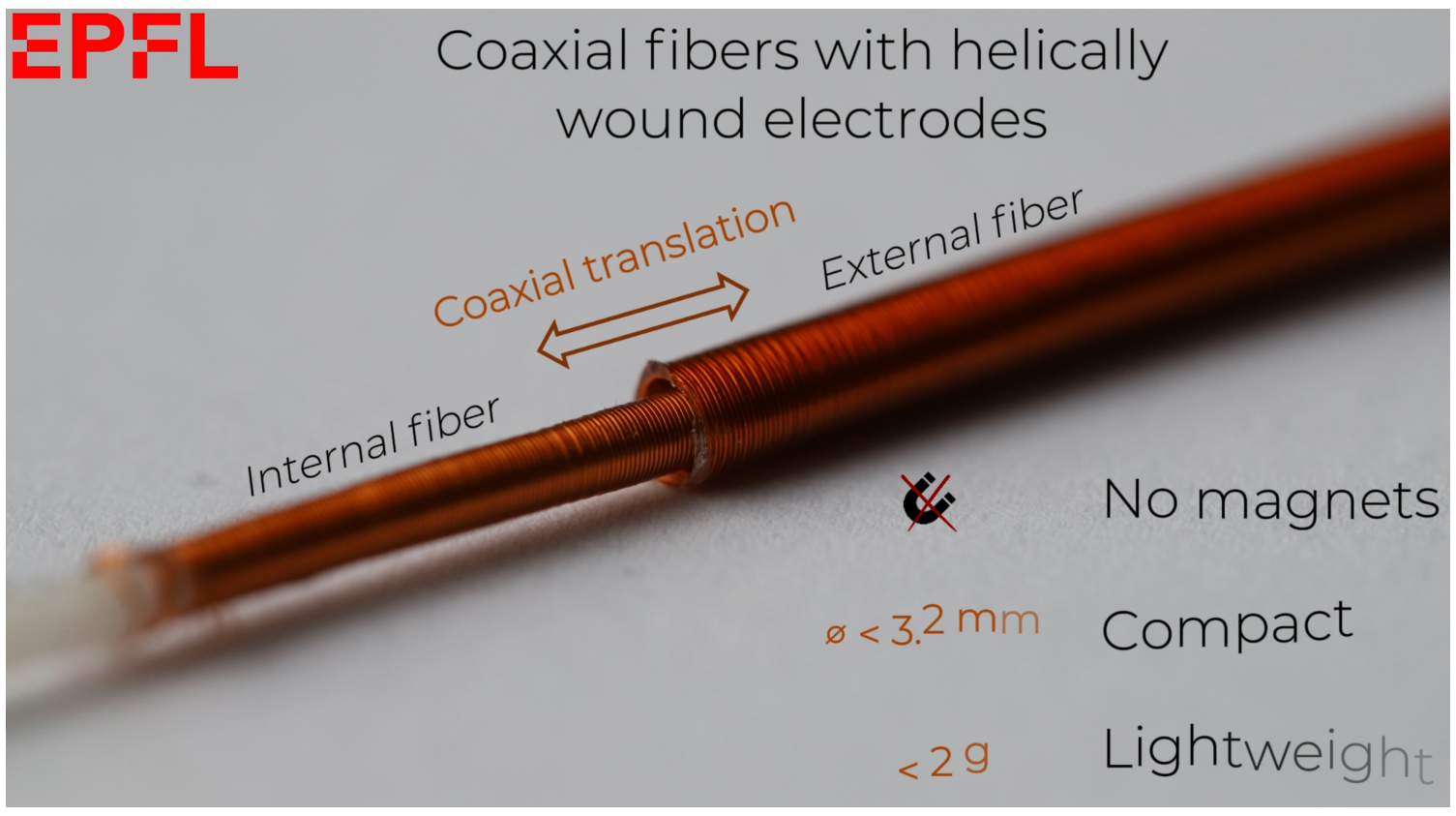

SI Video S1. Overview video of the FiberMotor concept and example applications

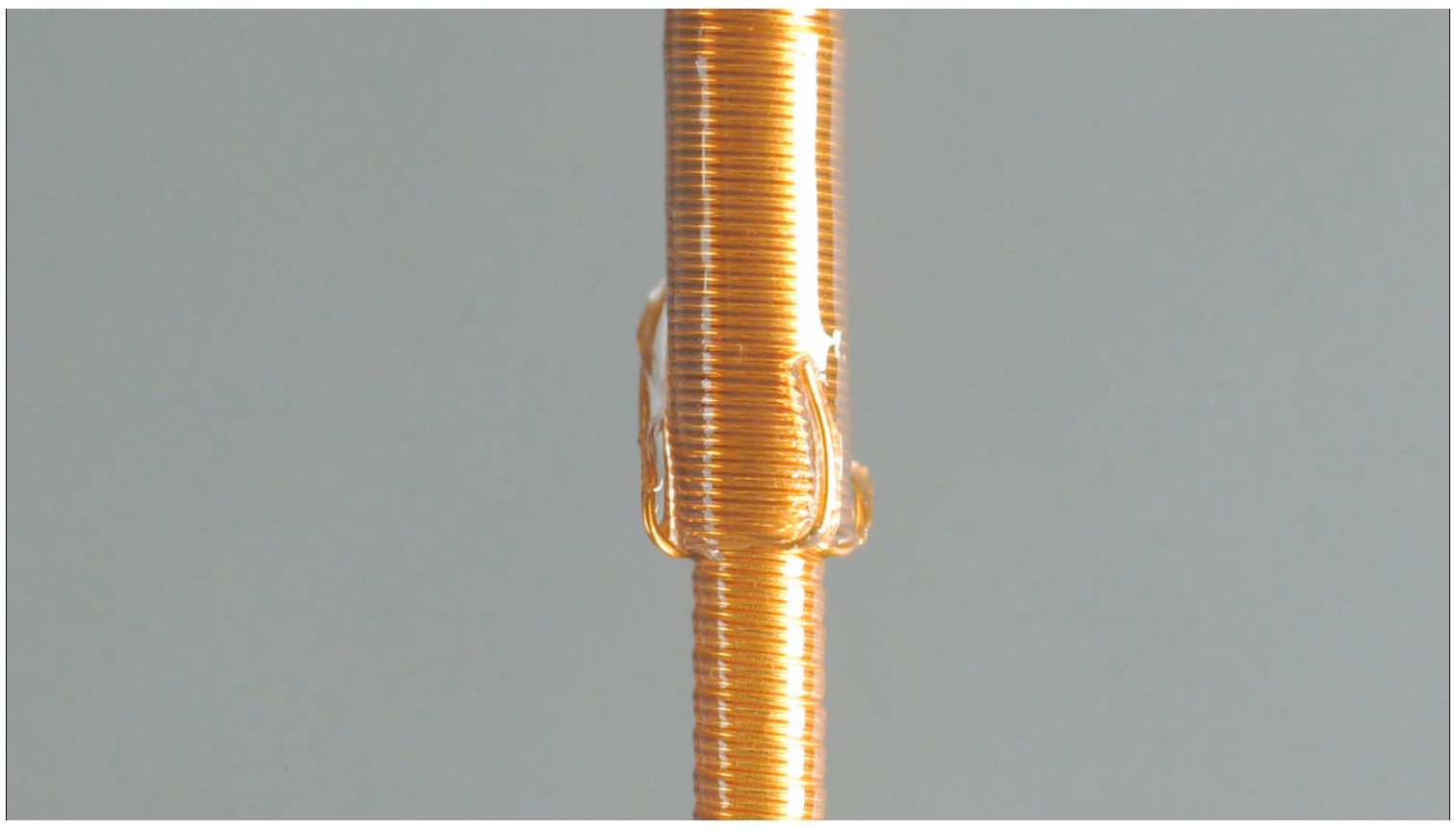

SI Video S2. Actuation at 0.5 Hz to show the step-by-step motion of a FiberMotor

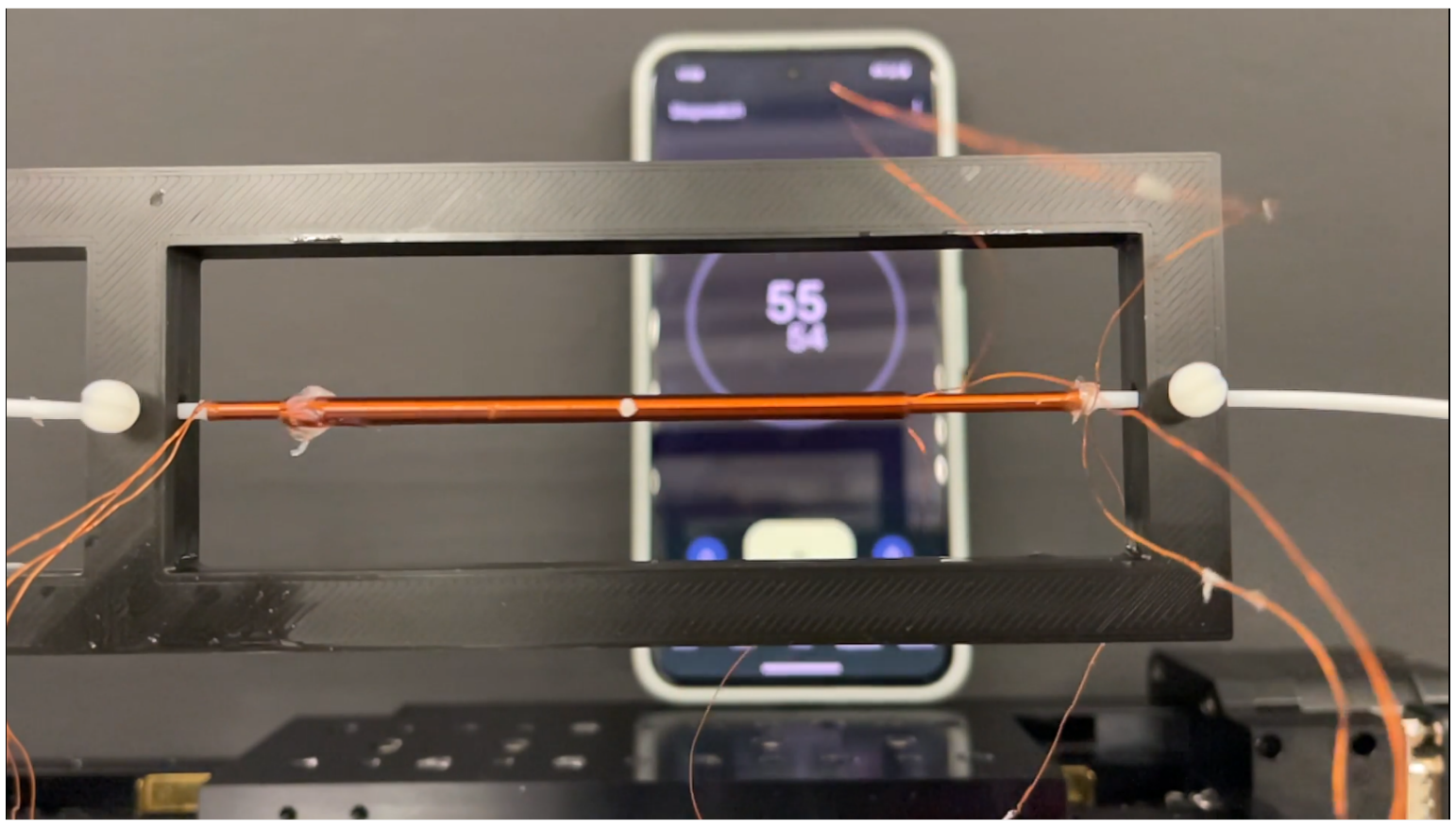

SI Video S3. Bidirectional fast motion at no load

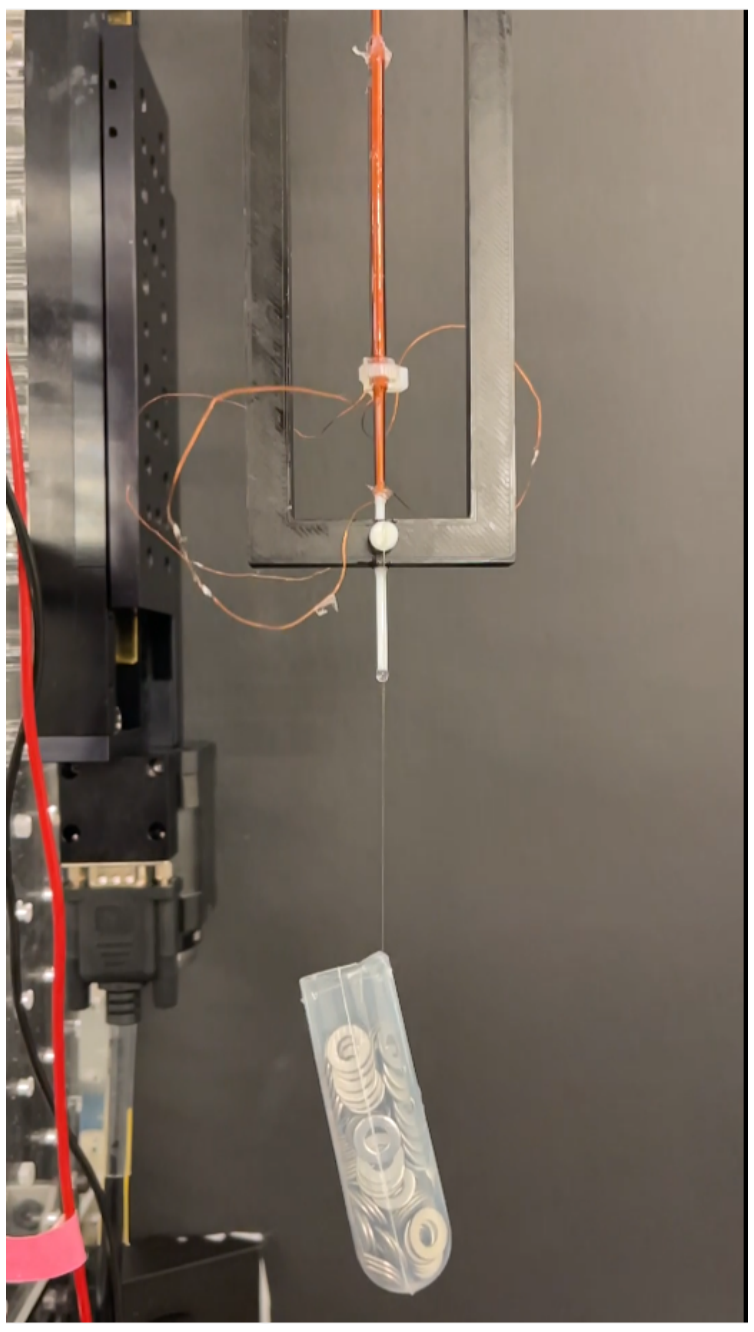

SI Video S4. FiberMotor lifting a 46 g payload

FiberMotor B1 lifting up a container loaded with 180 washers (458 mN).

FiberMotor B1 key metrics :
- Outer length 83 mm
- Copper wire diameter: 0.185 mm
- $OD_{outer\text{-}fiber}$: 3.16 mm

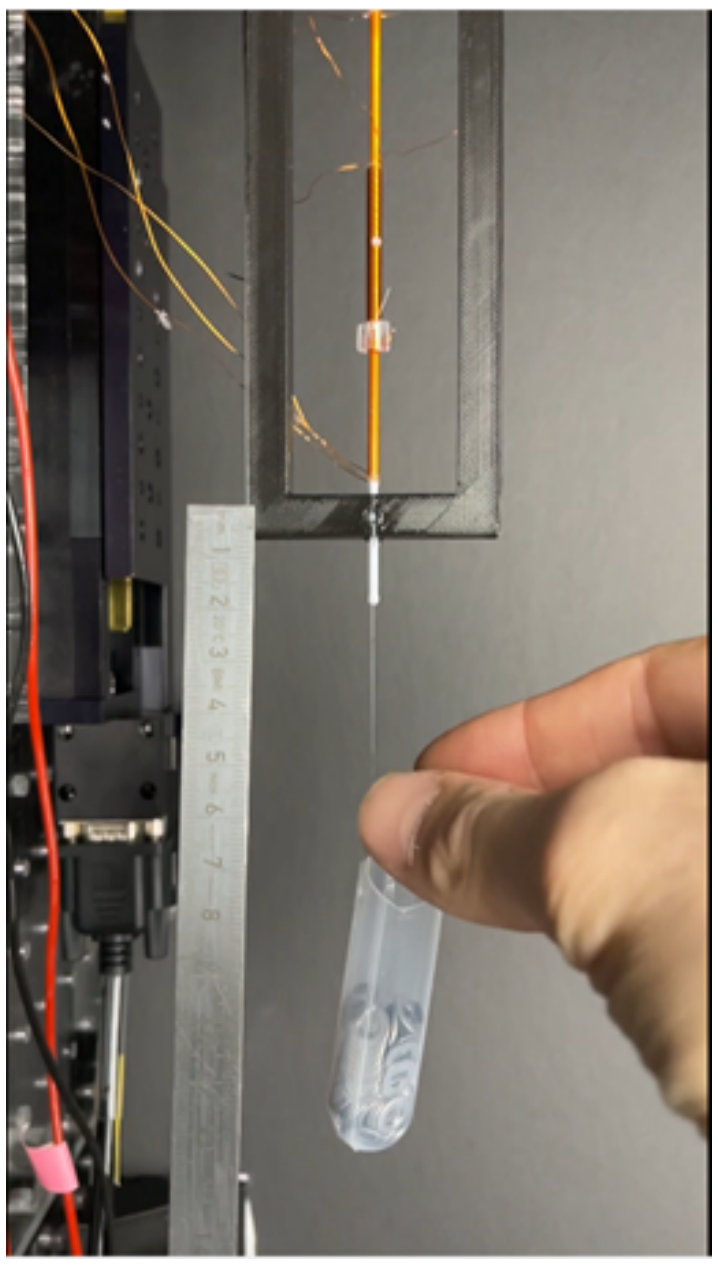

SI Video S5. FiberMotor holding a 24 g payload

Video of FiberMotor A1 holding 94 washers (247 mN).

FiberMotor A1 key metrics :
- Outer length 40 mm
- Copper wire diameter: 0.235 mm
- $OD_{outer\text{-}fiber}$: 3.24 mm

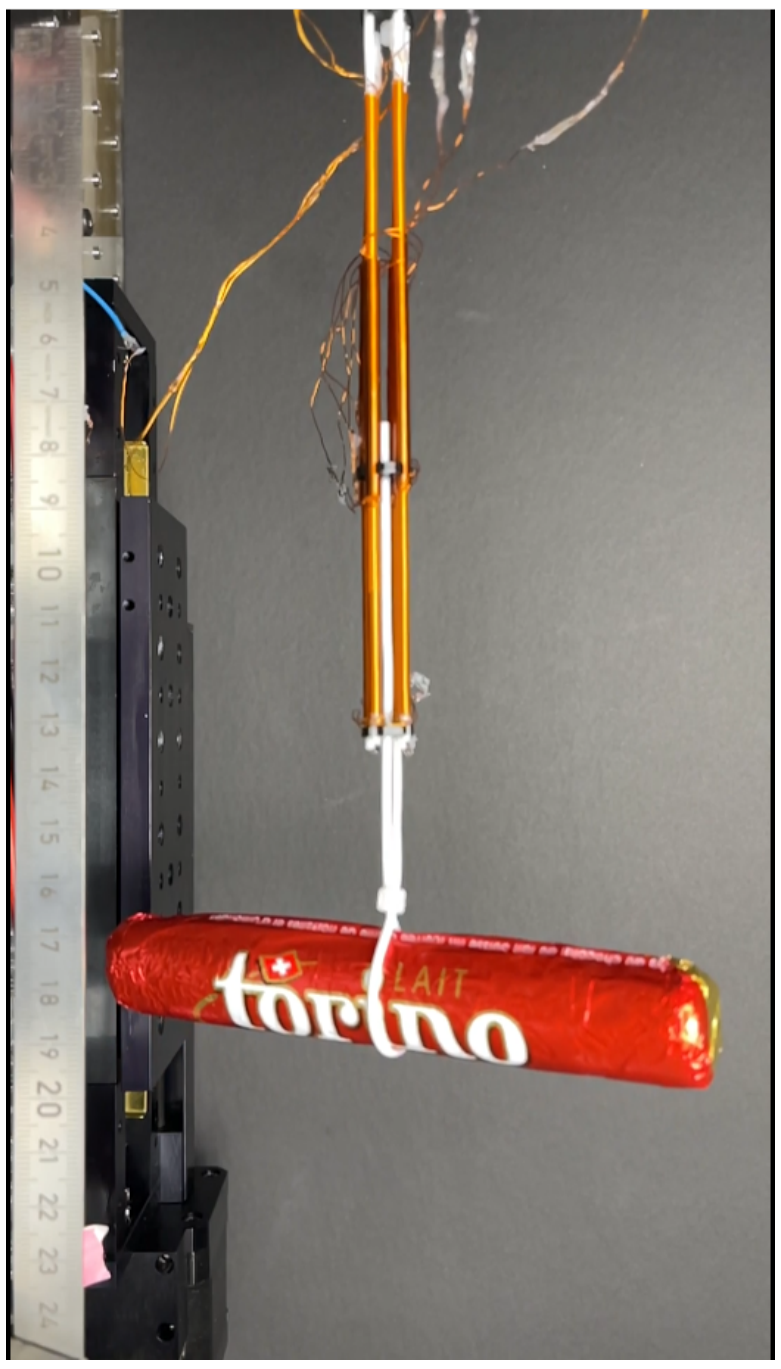

SI Video S6. Bundle of 4 FiberMotors i) lifting, ii) holding, iii) being backdriven, and iv) lowering a 46 g chocolate bar

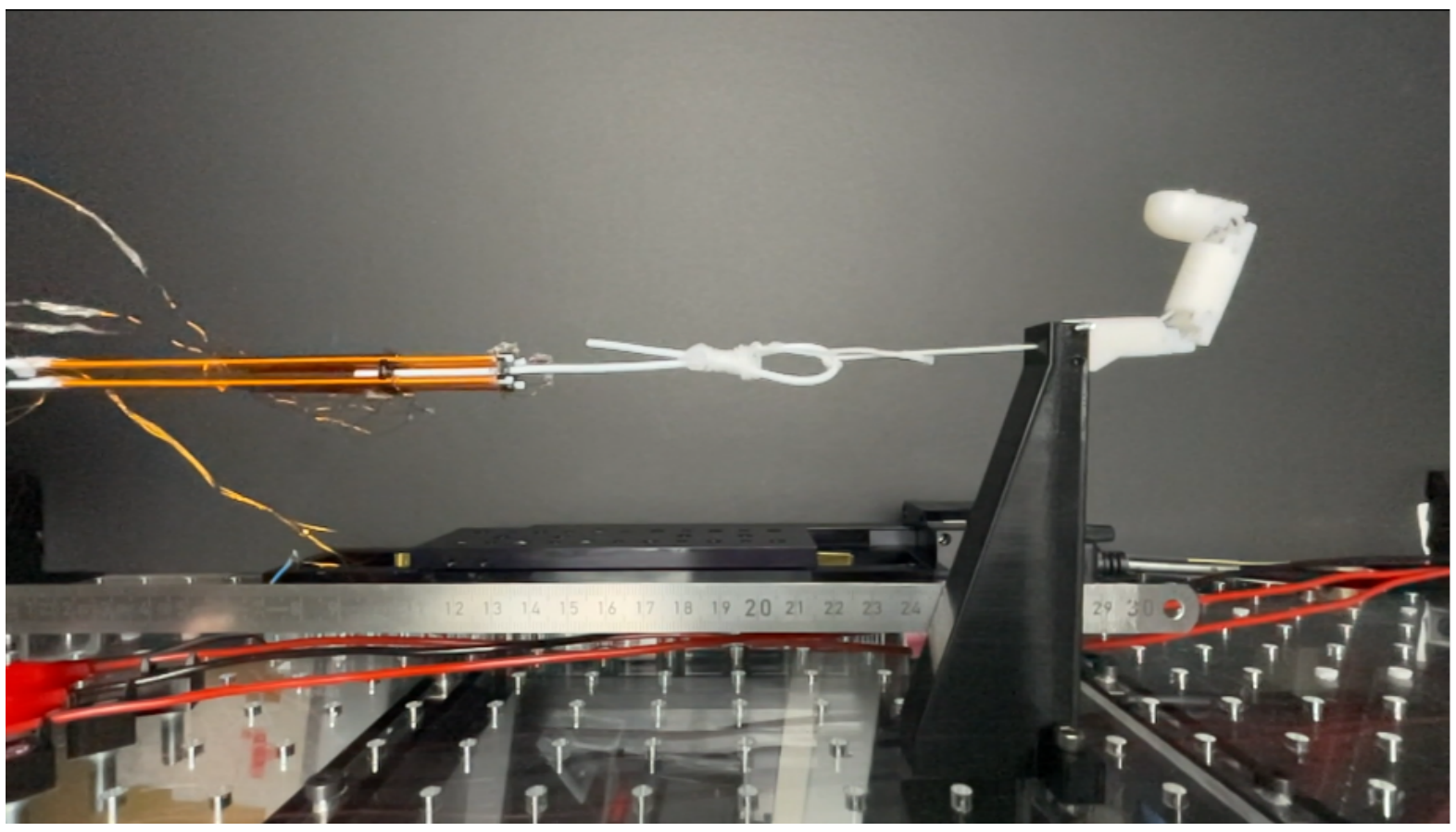

SI Video S7. Bundle of 4 FiberMotors pulling a tendon-driven 3D printed finger.

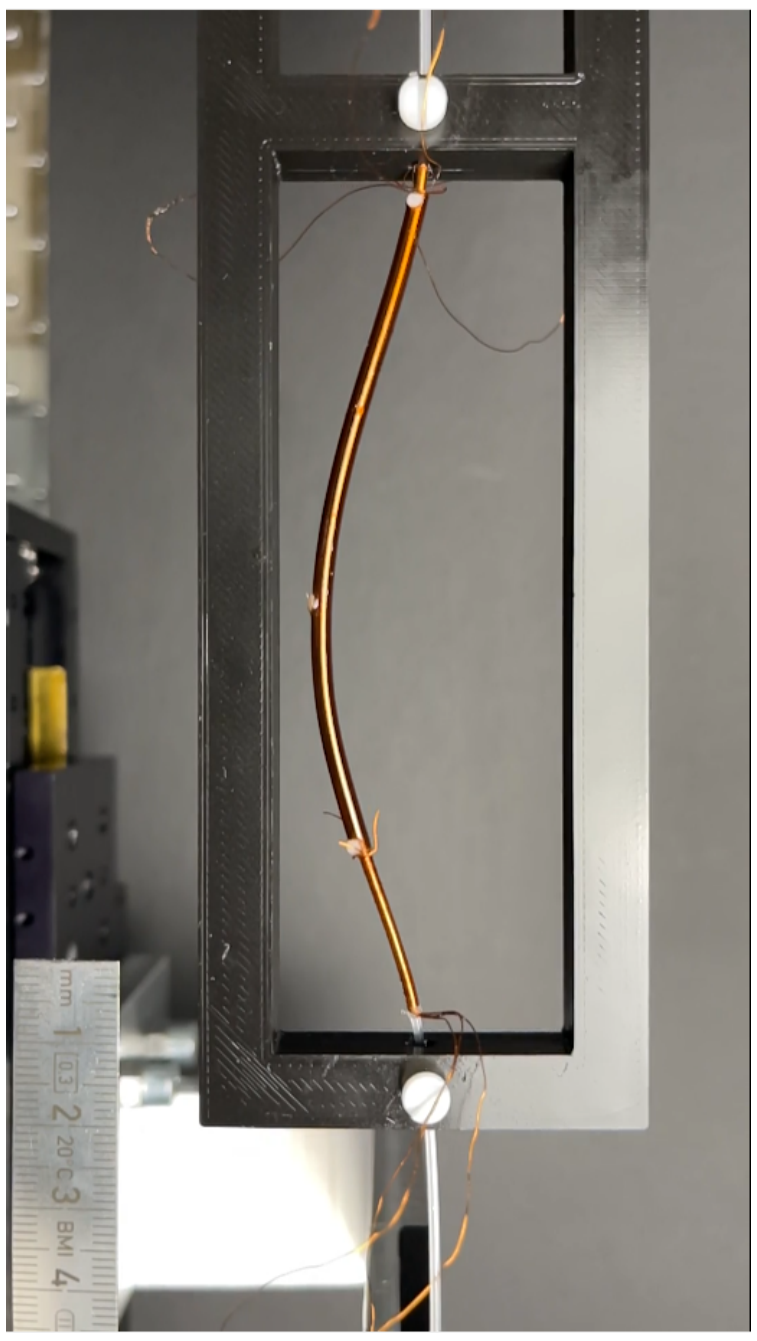

SI Video S8. The FiberMotor moves up and down while bent to a radius of curvature of 60 mm.

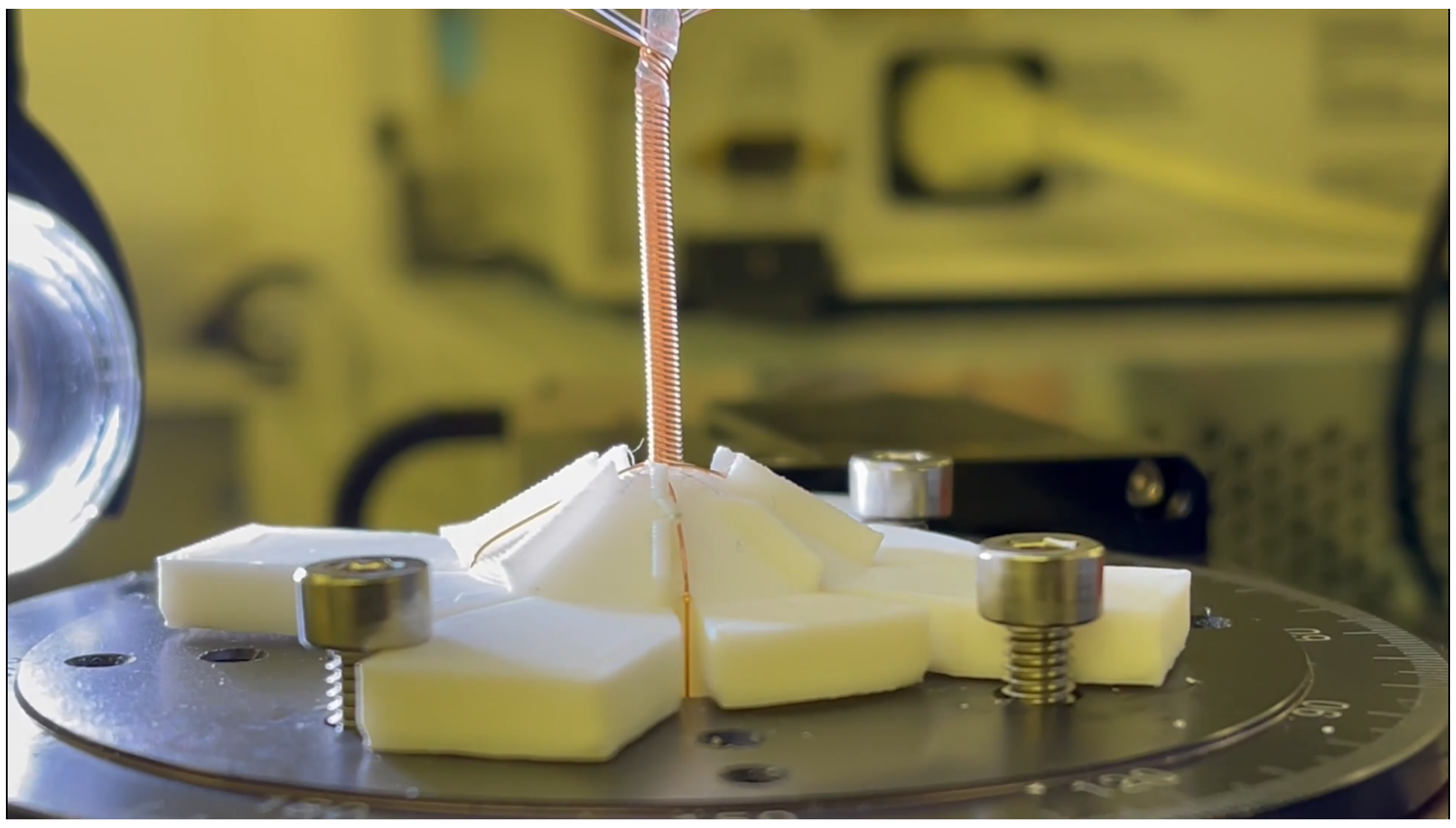

SI Video S9. MotorFiber winding process for an inner fiber, showing three enameled copper wires wound around the flexible inner core

SI- Table 1

Fiber-format actuator principle	Uni or bi- directional motion	Requires a preload	Continuous back and forth motion	Max strain or max stroke/length category	Time to move back and forth by 2 cm	References
**Flexible Sliding Electrostatic (FiberMotor)**	**Bi-directional**	**No**	**Yes**	**High** Stroke / longest length: 90%	**Fast** 0.25 s	This work
**Sliding Electrostatic**	**Not specified**	**No**	**Not specified**	**Not specified**	**Not specified**	Higuchi et al. (*45*)
**Compressive Electrostatic (DEA fiber)**	Uni directional	Yes	No	**Low** 1-10%	**Low** ~5-10 s	Kunze et al. (*17*) Kang et al. (*29*)
**Fluidic (McKibben)**	Uni directional	**No**	No	**Medium** 20%	**Moderate** ~1-5 s	Kilic Afsar et al. (*38, 39*)
**Fluidic, other**	Uni directional	Yes/ No	No	**High** 50% (250% at no load)	**Moderate** ~1-5 s	Phan et al. (*18*) Higueras et al. (*23*)
**Thermal (TCPA/SMA/LCE)**	Uni directional	Yes	No	**Medium** 30-60%	**Slow** >10 s	He et al. (*19*) Kim et al. (*21*) Zhang et al. (*33*) Haines et al. (*20*)
**Photonic**	Uni directional	Yes	No	**Medium** 60%	**Slow** >10 s	Iamsaard et al. (*22*)
**Moisture driven**	Uni directional	**No**	No	**High** 70%	**Slow** > 30s	Jia et al. (*39*)

SI Table 1. Overview of fiber-format actuators.

SI- Figures

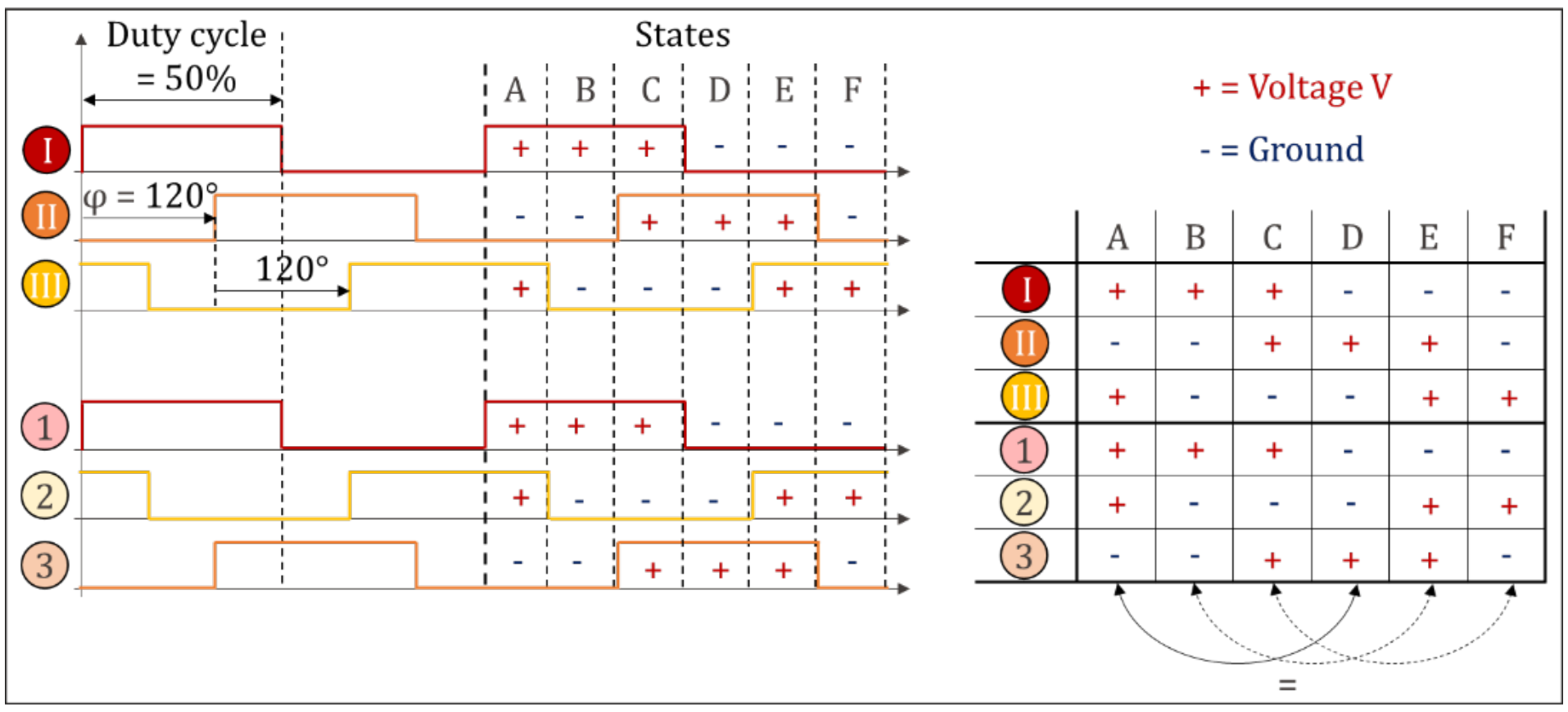

Fig. S1. Electrostatic drive states. Square unipolar drive signals with 50% duty cycle generated by the three-channel power supply for each pair of electrodes: Channel 1 – I/1, Channel 2 – II/3, and Channel 3 – III/2. Six distinct electrostatic configurations are defined (A, B, C, D, E, and F), corresponding to the combinations of high voltage and ground across the three channels. Symmetric configurations with inverted voltages produce equivalent electrostatic forces. For example, configuration A (Channels 1 and 3 at voltage V, Channel 2 grounded) is electrostatically equivalent to configuration D (Channels 1 and 3 grounded, Channel 2 at voltage V).

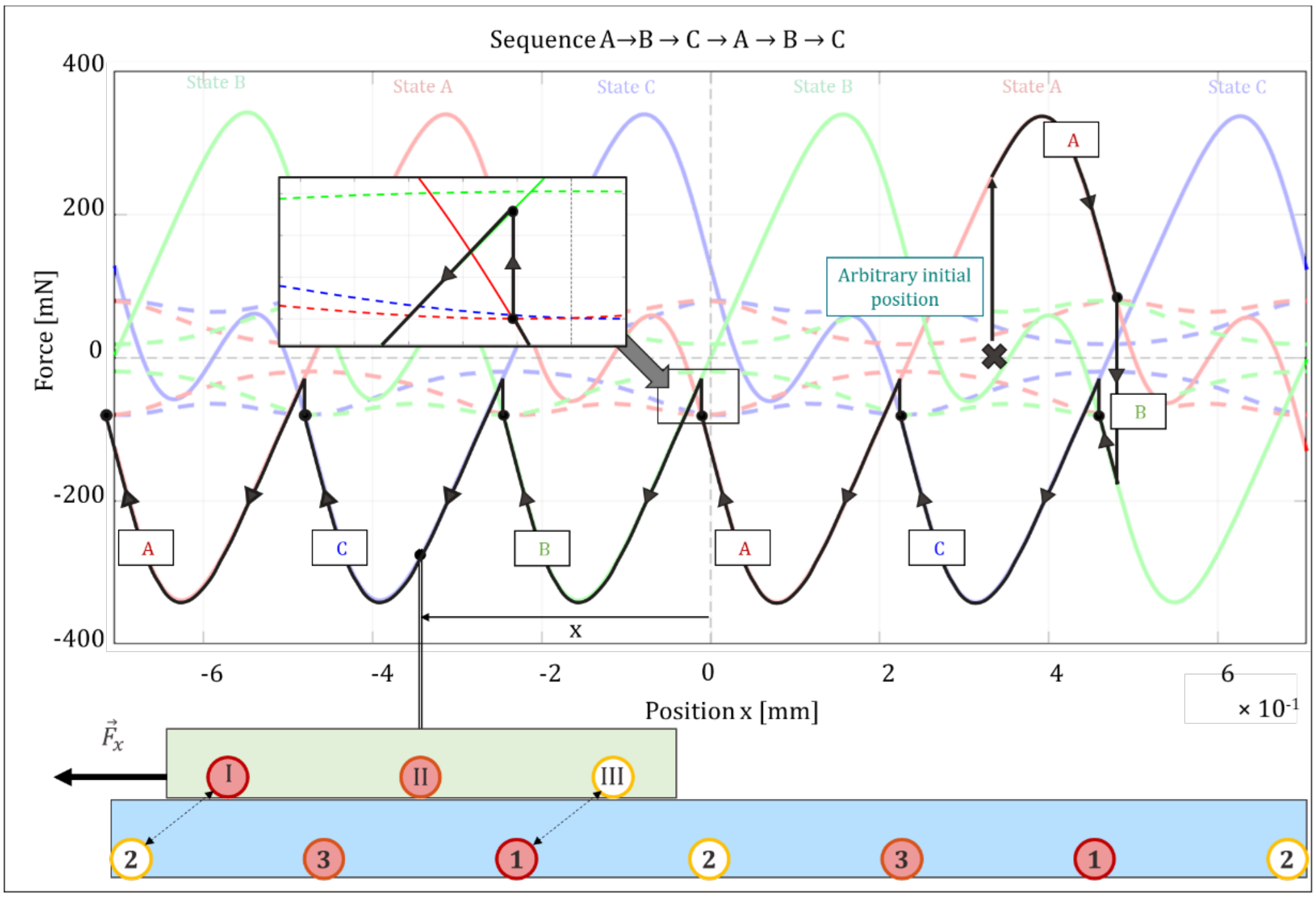

Fig. S2. Analytical plot of force generation and example of position for the FiberMotor. Plots of force vs position for configurations A, B, and C (color) with a step-by-step illustration of the actuation sequence (solid black line with arrows). Starting from an arbitrary position, drive state A is activated. Since $F_x > \mu F_y > 0$ in this position, the mobile fiber moves rightward first until it reaches the intersection point where $F_x = \mu F_y$, at which it stops. Drive state B is then activated, the force then goes $F_x < \mu F_y < 0$ moving then leftward. Drive state C is then activated producing similar conditions and continuing leftward motion, followed by drive states A, B, C, and so on. The intersections of the force plots are considered the points of maximum pulling force, representing the minimum force required to initiate a step. A zoom at the intersection is presented to show the first stop position at the intersection point $F_x = \mu F_y$ for drive state A and then when B is activated the transition with $F_x < \mu F_y < 0$ again to allow the motor to slide. A schematic of the motor was drawn below the plot as an example of position. Arrows between the electrodes represents main forces that illustrate the left sliding of the motor.

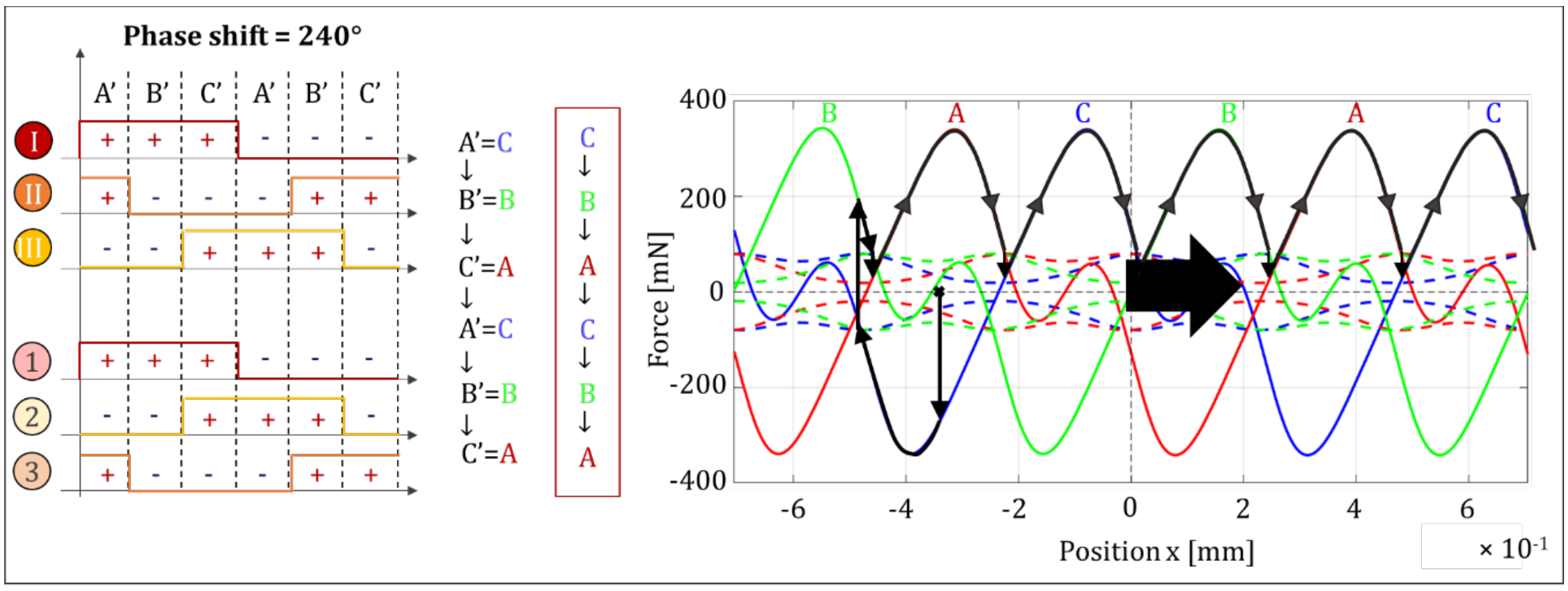

Fig. S3. Analytical plot of force generation for backward motion. When the phase shift is changed from 120° to 240°, we define new drive states A', B', C', D', E' and F'. Those new drive states correspond to a new sequence of the previous A, B and C drive states define for the phase shift = 120°. As shown in the figure, A', B', C', … is equivalent to C, B, A, C, B, A sequence. Using the same method as Fig 3.D, the motor moves rightward.

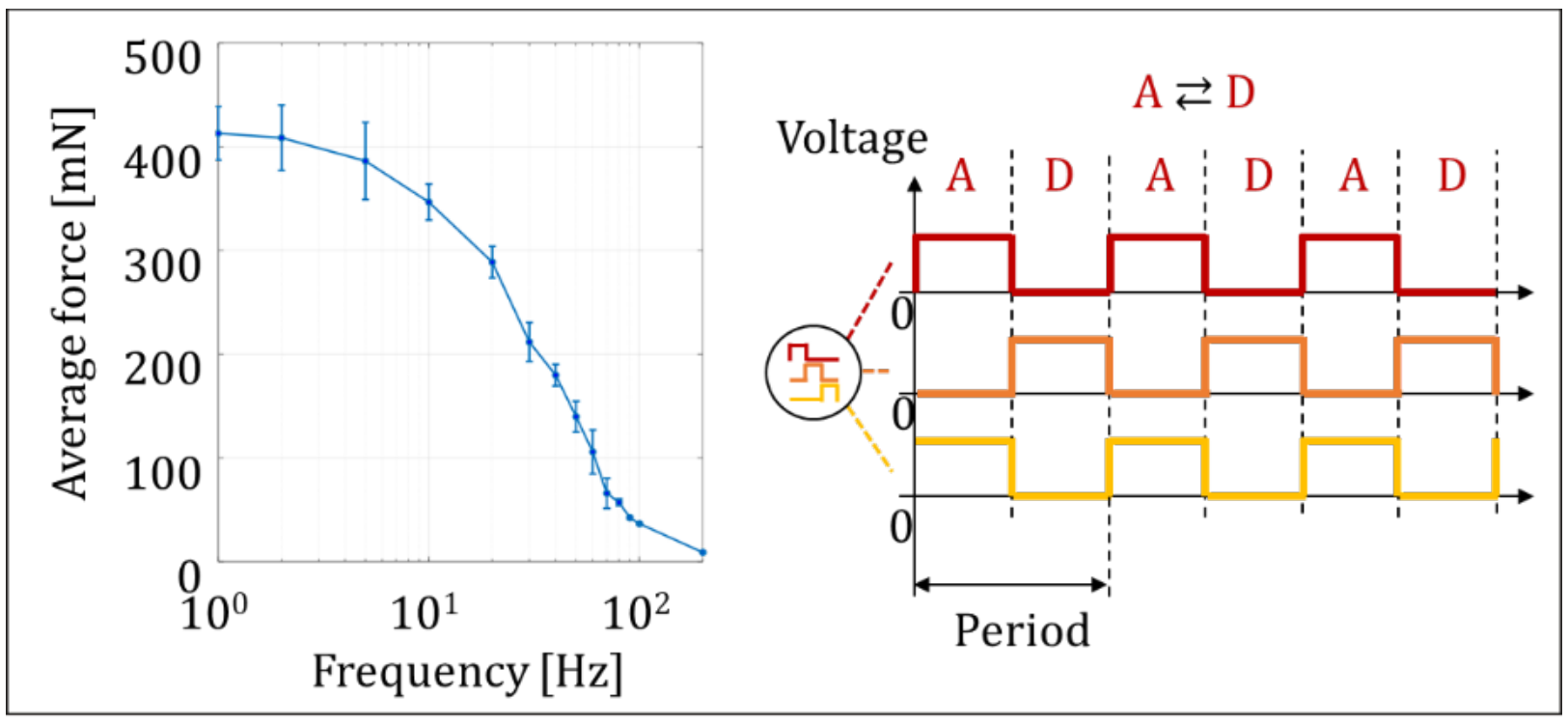

Fig. S4. *F*orce versus drive frequency. The force vs. drive frequency was measured using the same setup as for the clamped force characterization. The outer fiber is clamped to the linear motor and fixed at the position of maximum force at drive state A. The inner fiber is again attached to the force sensor. The drive signal we generated correspond to alternate drive state A and its symmetric drive state D.

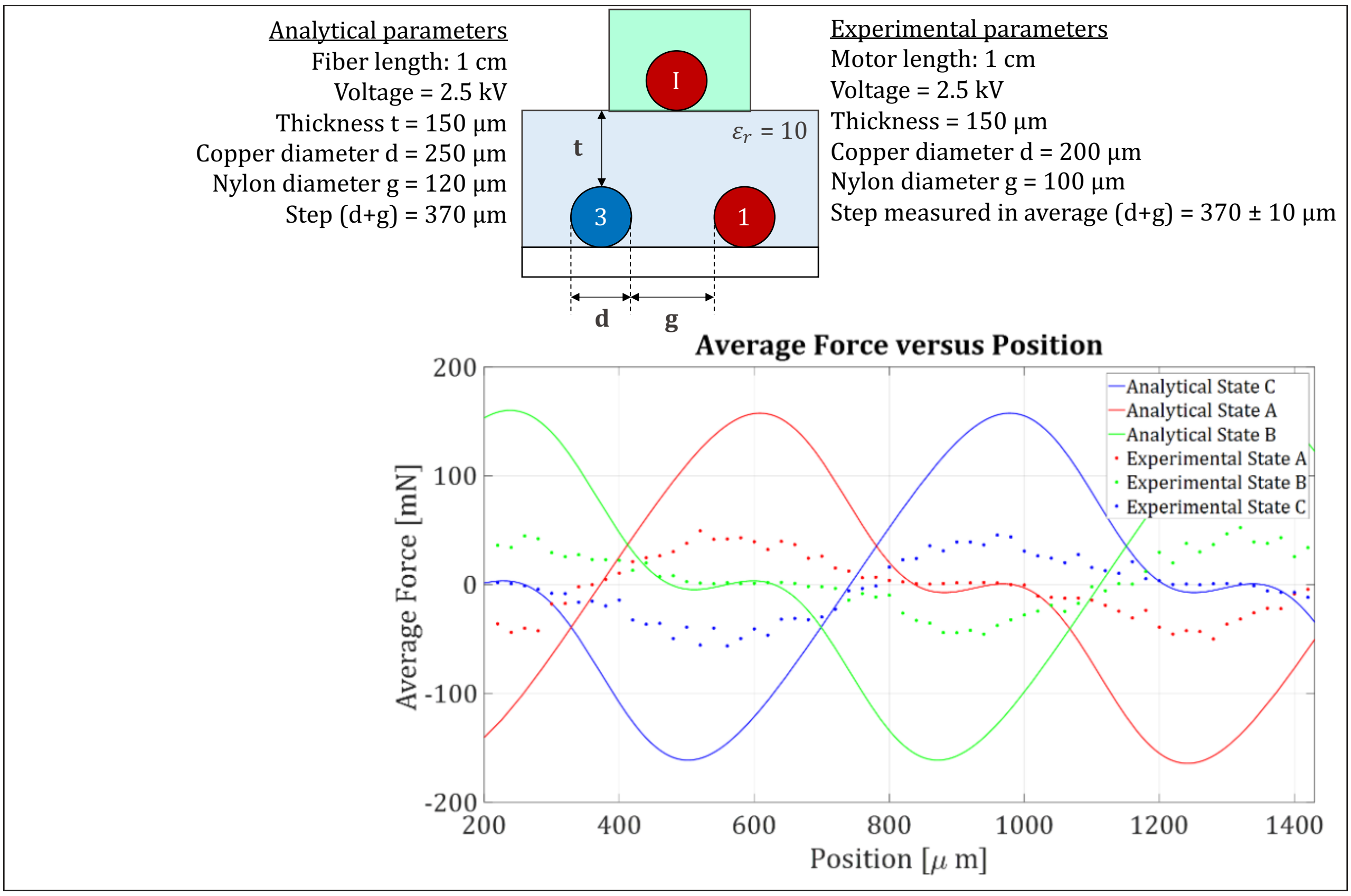

Fig. S5. Average Force vs. position. Top: schematic of the motor with values of analytical parameters used in the model and experimental dimensions. Bottom: Unscaled analytical model of "average force versus position" curve plotted with the experimental measurements. The amplitude of the plots would match if the analytical predictions were scaled by a factor of 0.36.

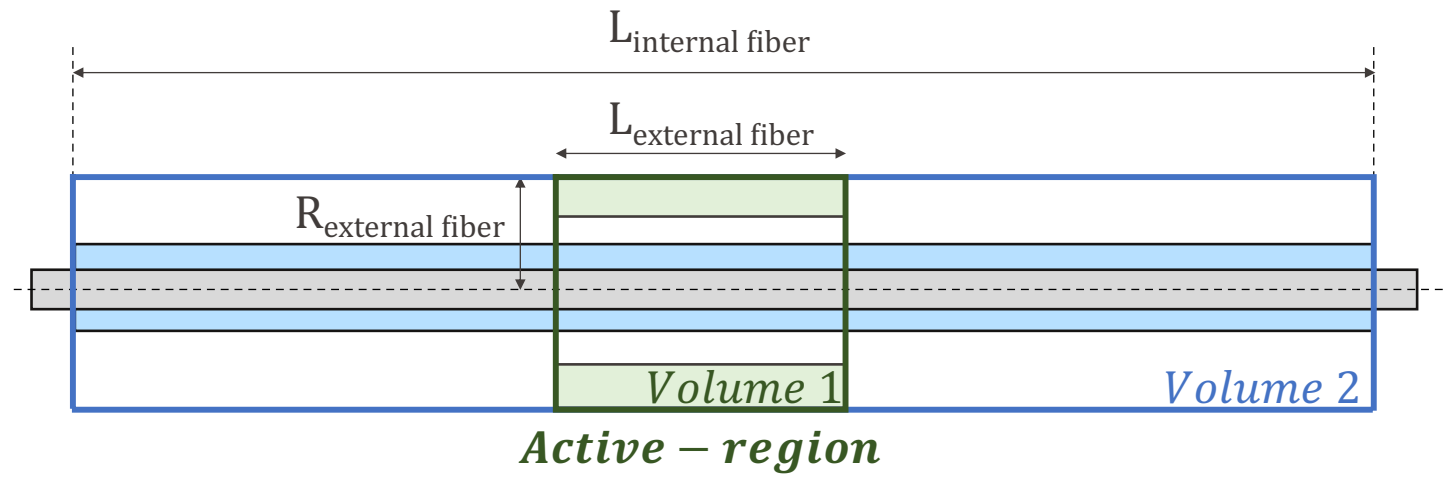

Example:

$L_{internal\ fiber} = 40$ mm

$L_{external\ fiber} = 100$ mm

$R_{external\ fiber} = 3{,}25$ mm

Payload weight for 12 washers
$= 1.719\ g\ (empty\ weight\ of\ the\ containers) + 12 * 0.250\ g\ (weight\ of\ 1\ washer)$
$= 4.719\ g$

Pulling displacement $= 25.61\ mm$

Volume

$$Volume\ 1 = L_{external\ fiber} * \pi * R^2_{external\ \ fiber} = 40 * 10^{-3}\ [m] * \pi * (3.25/2 * 10^{-3})^2\ [m^2] = 0.33\ * 10^{-6}\ [m^3]$$

$$Volume\ 2 = L_{internal\ fiber} * \pi * R^2_{external\ \ fiber} = 100 * 10^{-3}\ [m] * \pi * (3.25/2 * 10^{-3})^2\ [m^2] = 0.83\ * 10^{-6}\ [m^3]$$

Work density

$$Payload\ force = Payload\ weight * 9.81 = 46.3\ mN$$

$$Work = Payload\ force\ * Pulling\ displacement = 46.3\ * 25.61 = 1185.57\ \mu J = 1.19 * 10^{-3}\ J$$

$$Work\ density_{\ Volume\ 1} = \frac{work}{Volume\ 1} = \frac{1.19 * 10^{-3}}{0.33 * 10^{-6}} = 3600\frac{J}{m^3} = 3.6\frac{kJ}{m^3}$$ → Work density used in the Fig. 6

$$Work\ density_{\ Volume\ 2} = \frac{work}{Volume\ 2} = \frac{1.19 * 10^{-3}}{0.83 * 10^{-6}} = 1434\frac{J}{m^3} = 1.4\frac{kJ}{m^3}$$

Fig. S6. Work density computation. We compare in this figure the work density for two different possible definitions of the active volume. The first definition of volume includes only the active-region volume: $0.33\times10^{-6}\ m^3$. The second definition corresponds to the total volume swept by the motor during full stroke operation: $0.83\times10^{-6}\ m^3$. The work density presented in Fig 6 is computed using the first volume definition, namely only the active-region volume.

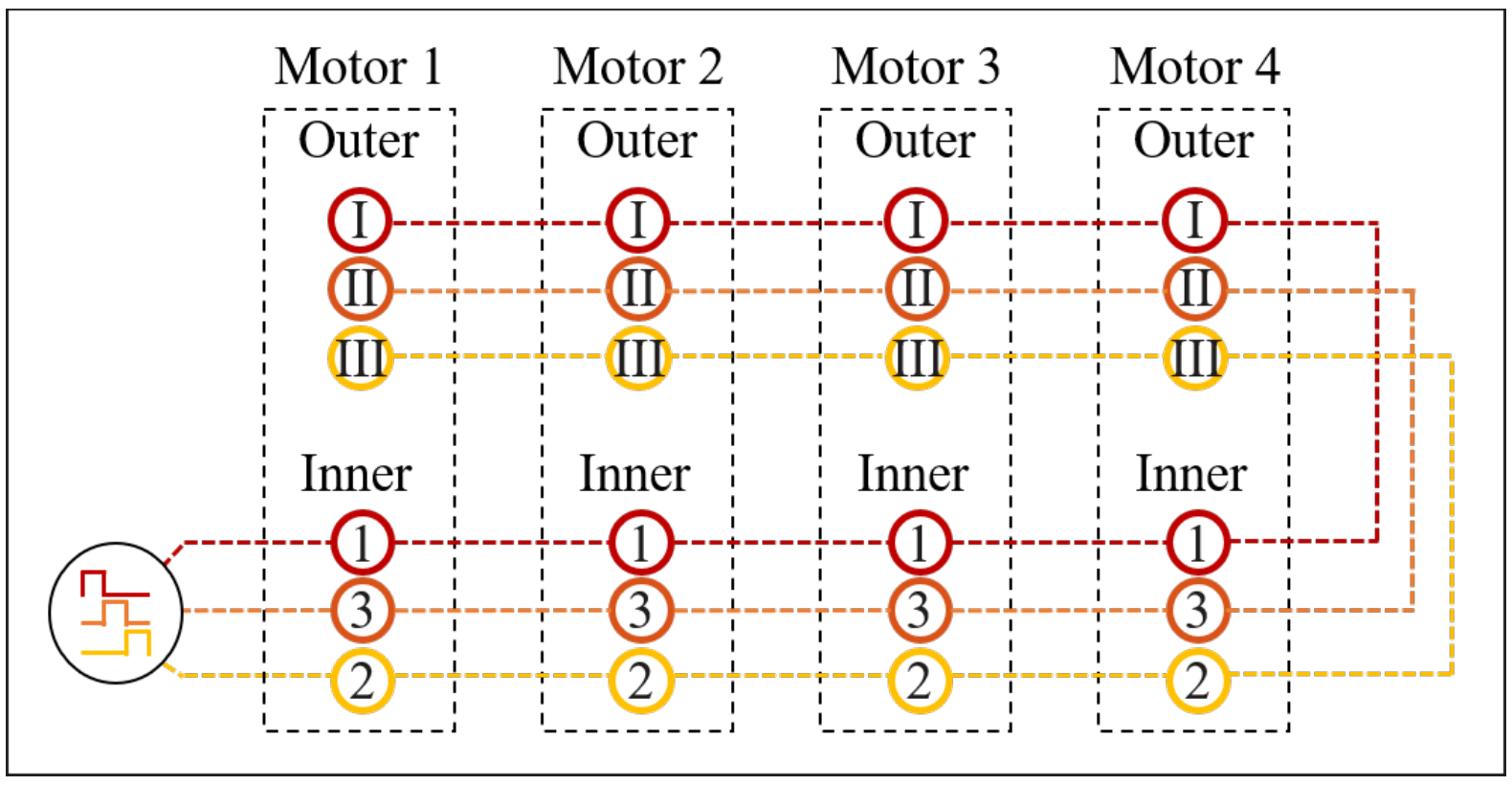

Fig. S7. Schematic of the electrical connections used for bundling four FiberMotors. This diagram illustrates the series wiring configuration described in Section 4.5. The high-voltage supply is applied to the inner electrodes of Motor 1, after which the inner electrodes are connected sequentially (inner 1 → inner 2 → inner 3 → inner 4). The circuit is then closed by linking the inner electrode of Motor 4 to the outer electrode of Motor 4, followed by the outer electrodes connected in reverse sequence (outer 4 → outer 3 → outer 2 → outer 1). This series-series linkage ensures that all four FiberMotors receive the same drive signal and activate simultaneously.

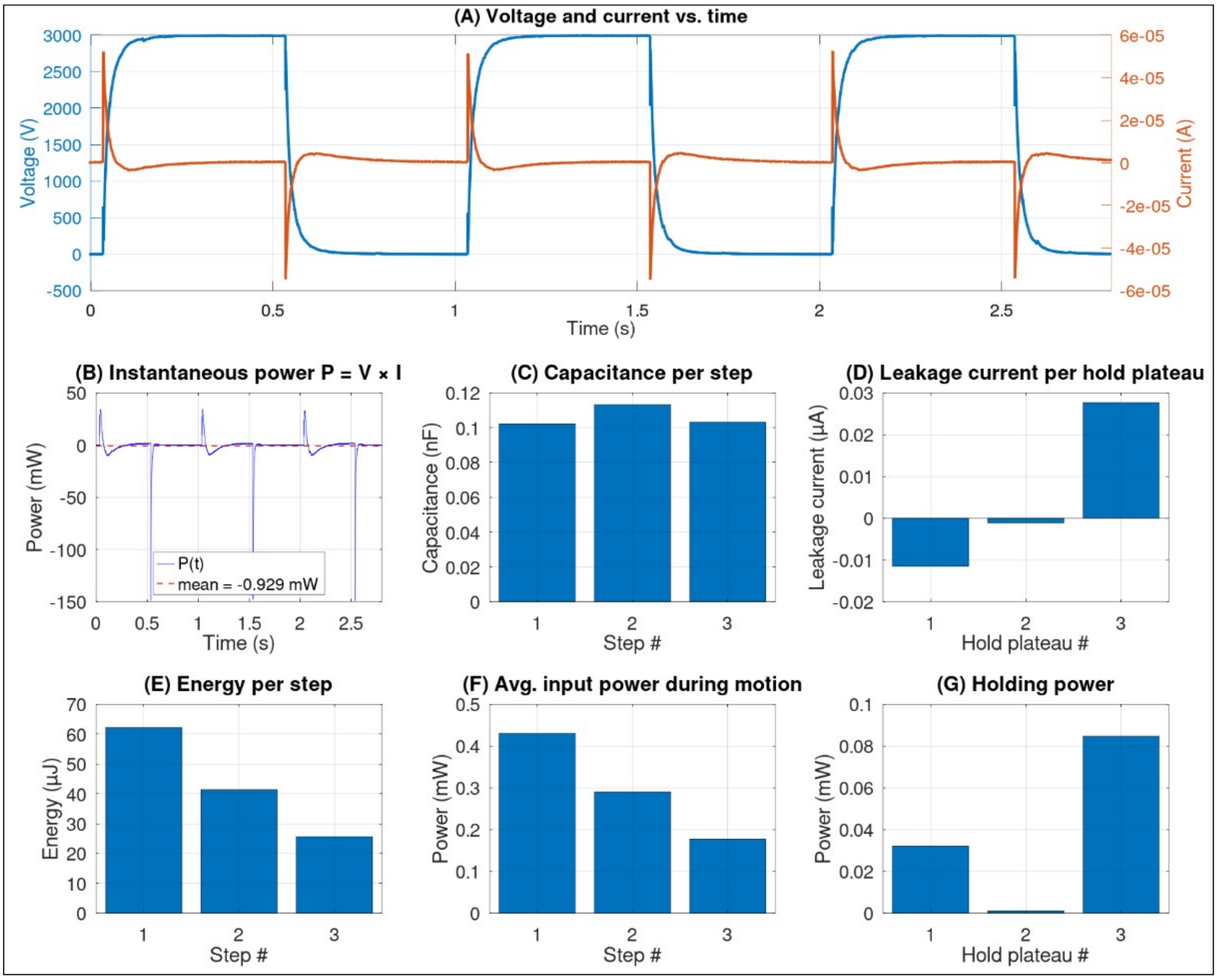

Fig. S8. Electrical characterization derived from a Voltage and current waveform for one complete drive phase at 1 Hz. (A) Voltage and current vs. time. (B) Instantaneous electrical power $P(t) = V(t)\,I(t)$. The dashed red line is the time-average of $P(t)$ over the entire record. (C) Capacitance estimated for each detected charging transient (step). Transients are detected where |I(t)| exceeds 3% of the peak current; a segment is kept as a "step" only if the voltage rises from below 599 V to above 2694 V. Charge Q is obtained by integration of I(t). Capacitance C = Q / deltaV, with deltaV is the voltage change over the transient. (D) Leakage current for each detected steady high-voltage plateau (hold), defined as periods where V > 2694 V and |I(t)| stays below the transient threshold for at least 30 ms. Leakage current is the time-average of I(t) over each plateau. (E) Electrical energy consumed during each charging step, computed as the integral of $P(t) = V(t)\,I(t)$ over the duration of that transient. (F) Average electrical input power during each charging step, computed as the energy in panel (E) divided by the duration of that transient. (G) Holding power for each hold plateau, computed as the time-average of P(t) over that plateau (coherent with leakage current).
